# *Oviductus Ranae* Protein Hydrolysate (ORPH) Ameliorates CUMS‐Induced Depressive‐Like Behaviors in Mice via Modulation of Gut Microbiota and Metabolic Signatures: An Integrated Multi‐Omics Investigation

**DOI:** 10.1002/fsn3.72572

**Published:** 2026-11-13

**Authors:** Mengmeng Liu, Jingyi Niu, Yukang Miao, Wentao Guo, Xiaowei Huang, Hongyan Chen, Zhe Lin, Guangfu Lv

**Affiliations:** ^1^ Changchun University of Chinese Medicine Changchun China; ^2^ The Affiliated Hospital to Changchun University of Chinese Medicine Changchun China

**Keywords:** chronic unpredictable mild stress, depression, gut microbiota, *Oviductus Ranae*, *Oviductus Ranae* protein hydrolysate

## Abstract

Depression constitutes a significant global health burden, manifesting as persistent low mood, anhedonia, and behavioral disturbances. The pathogenesis of depression is multifactorial, involving hypothalamic–pituitary–adrenal (HPA) axis dysregulation, monoaminergic neurotransmitter abnormalities, and neuroinflammatory processes. *Oviductus Ranae* protein hydrolysate (ORPH) is an enzymatically hydrolysed extract derived from the dried oviducts of female *Rana dybowskii Günther* (*Oviductus Ranae*, OR). This esteemed traditional Chinese medicine, indigenous to Northeast China, has been utilized as a valuable tonic for centuries. Previous in vivo investigations have demonstrated the anti‐neuroinflammatory effects of OR. Chronic unpredictable mild stress (CUMS) is widely used to establish a mouse model of depressive‐like phenotypes. This study aimed to investigate whether ORPH could alleviate CUMS‐induced depressive‐like behaviors in mice and explore its underlying mechanisms from the perspective of gut microbiota and metabolic profiles. A CUMS paradigm was employed to establish a murine model of depression, and the effects of 0.2, 0.4, or 0.8 g/kg ORPH on depressive‐like behaviors were systematically evaluated. Biochemical analyses were conducted to quantify inflammatory mediators, neurotransmitters, and HPA axis‐associated hormones in murine serum and brain tissue. Multi‐omics approaches, encompassing brain tissue metabolomics, fecal metabolomics, and gut microbiota sequencing, were utilized to elucidate key metabolic constituents and signaling pathways implicated in the regulation of cerebral and gut microbial communities. In vivo experiments were conducted to investigate the effects of microbiota depletion and blockade of key metabolites on the therapeutic efficacy of ORPH. Administration of ORPH at doses of 0.2, 0.4, or 0.8 g/kg markedly ameliorated depressive‐like behaviors in CUMS‐exposed mice. ORPH increased the sucrose preference rate, reduced immobility time in the FST and TST, and shortened the escape latency in the MWM of CUMS mice. Biochemical assays revealed that ORPH attenuated serum and cerebral concentrations of pro‐inflammatory cytokines (IL‐6, IL‐1β, and TNF‐α), reduced circulating ACTH and CRH levels, decreased cerebral CORT content, and elevated cerebral 5‐HT and NE levels. Moreover, ORPH significantly mitigated hippocampal tissue injury and apoptosis. Metabolomic analyses identified therapeutic modulation of pathways such as sphingolipid and tryptophan metabolism, while gut microbiota profiling highlighted the enrichment of these taxa, including *Lactobacillus*. The protective effect of ORPH was obviously weakened after microbiota depletion, and similar attenuation was observed upon inhibiting tryptophan hydroxylase (TPH) activity and sphingosine kinase (SphK). These findings indicate that ORPH may exert favorable therapeutic potential against CUMS‐induced depressive‐like phenotypes, possibly through modulation of dysregulated sphingolipid and tryptophan metabolism, which could contribute to the alleviation of neuroinflammation linked to gut microbiota dysbiosis.

## Graphical abstract

*Oviductus Ranae* protein hydrolysate (ORPH) ameliorates CUMS‐induced depressive‐like behaviors in mice, accompanied by altered gut microbiota and tryptophan/sphingolipid metabolism.

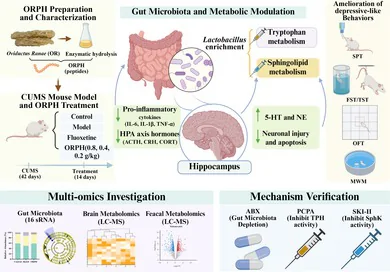

## 1 Introduction

Depression is a condition characterized by persistent low mood and anhedonia, and is one of the most common mental and behavioral disorders worldwide. It is caused by a variety of factors, including family history, substance abuse, physiological and pathological factors, chronic health issues, and significant life changes (Bowes et al. 2016). Depression has a significant impact on global public mental health. Patients with depression may exhibit symptoms such as pessimism, decreased attention, reduced energy, and sleep disturbances, and in severe cases, may even exhibit suicidal tendencies (Hammen 2018). According to the World Health Organization (WHO), there are approximately 380 million people worldwide suffering from depression, and its high incidence and disability rates make it a significant challenge to global public health (Rong et al. 2025). Researchers have proposed various hypotheses regarding the pathogenesis of depression, such as the neuroplasticity hypothesis, the monoamine neurotransmitter hypothesis, the HPA axis dysfunction hypothesis, and the neuroinflammation hypothesis (Liu et al. 2023). However, depression is not a disease caused by a single factor; it is a complex multi‐system disease, and its pathogenesis cannot be fully understood by relying on any single hypothesis. In recent years, with the continuous development and understanding of gut microbiota diversity and biological functions, the exploration of gut microbiota as a therapeutic target for diseases has garnered increasing attention (Zhao et al. 2026). The regulation of gut microbiota for disease treatment has gradually been applied and developed, with numerous studies indicating a strong association between depression and gut microbiota (Zainal Abidin et al. 2025; Zhu et al. 2022). The gut microbiota, often referred to as the ‘second brain’ of humans, plays a crucial role in neurotransmission, oxidative stress, and inflammatory responses (Rasmus and Kozłowska 2023), and is closely linked to the onset and progression of depression (Alli et al. 2022). Existing studies have reported that the diversity and abundance of the gut microbiota in depression patients have undergone significant changes, and effective regulation of the gut microbiota can treat depression (Chang et al. 2022). Currently, commonly used antidepressant medications include serotonin reuptake inhibitors, amitriptyline, and monoamine oxidase inhibitors (Boku et al. 2018). However, these medications have limitations in efficacy due to issues such as delayed onset of action, low efficacy rates, significant side effects, and limited modulation of inflammation and microbiota dysbiosis (Tian et al. 2022). Therefore, identifying milder and more effective antidepressant medications has become a new research objective. In contrast, enzyme‐extracted herbal medicine extracts are rich in small‐molecule active peptides and bioactive substances, making them easier to absorb. They exhibit excellent bioavailability and have the potential for multi‐target regulation, effectively acting on the central nervous system.

*Oviductus Ranae* (OR) is the dried oviduct of the female *Rana dybowskii Günther*, a species of frog, and is a valuable traditional Chinese medicine (Pharmacopoeia of the People's Republic of China 2025). It has the effects of tonifying the kidneys, nourishing essence, and moisturizing the lungs, and has traditionally been used for anti‐fatigue and tonifying and regulating purposes. It has potential neuroprotective effects and has shown efficacy in improving depressive mood, earning it the reputation of ‘tonic soft gold’ (Zhang et al. 2019). Modern pharmacological studies have also confirmed the various pharmacological activities of OR, including enhancing memory, anti‐aging, anti‐anxiety, boosting immunity, and regulating blood lipids (Gongqing and Yikun 2025; Zhang et al. 2018). In our previous research, we found that OR has therapeutic effects on depression, but the underlying mechanisms remain unclear (Weijia et al. 2025). In our previous work on the antidepressant effects of OR, we also found that OR potentially modulates the gut microbiota in depression‐model mice. On this basis, we hypothesized that OR could ameliorate depressive‐like behaviors in depressed mice via regulating gut microbiota. Therefore, the present study focuses on the gut microbiota‐modulating effects of *Oviductus Ranae* protein hydrolysate (ORPH). Integrating multi‐omics approaches, we used the chronic unpredictable mild stress (CUMS) method to establish a mouse model of depression, assessed depressive‐like behavior, measured levels of inflammatory factors, neurotransmitters, and HPA axis‐related hormones in brain tissue and serum, and analyzed differences in fecal tissue metabolites to examine changes in the gut microbiota of mice. The study explores the molecular mechanisms by which ORPH improves depression from the three aspects of ‘microbiota‐inflammation‐metabolism,’ providing scientific evidence for the development of novel multi‐target antidepressant drugs.

## 2 Materials and Methods

### 2.1 Materials and Reagents

OR is provided by Tonghua Ding Shen Pharmaceutical; Fluoxetine hydrochloride capsules are purchased from Shanghai Pharmaceutical; Pepsin and trypsin are purchased from Beijing Solabio Biotechnology; The CORT, ACTH, CRH, TNF‐α, IL‐1β, IL‐6, 5‐HT, and NE kits are purchased from Jiangsu Enzyme Immunoassay; PCPA, SKI‐II are purchased from MedChemExpress; Ampicillin sodium salt is purchased from Biosharp; Metronidazole, neomycin sulfate, and vancomycin hydrochloride are purchased from Aladdin.

### 2.2 ORPH Sample Preparation and Component Analysis

The dry OR powder was added with citric acid with pH 4.5 at the ratio of solid to liquid 1:100, soaked for 36 h, homogenized, then added with 2000 mL distilled water, boiled for 2 h, cooled, adjusted the pH to 3.0 ± 0.2 with 10 mmol/L HCl solution, and added pepsin at the ratio of enzyme to substrate 1:2000. Enzymolysis treated at 37°C for 2 h; after enzymolysis, the sample was boiled for 15 min, and the enzyme was inactivated. The pH was adjusted to 10.0 ± 0.2, trypsin was added according to the enzyme to substrate ratio of 1:100, enzymolysis treated at 55°C for 6 h; after enzymolysis, the sample was boiled for 15 min, and the enzyme was inactivated. The pH was adjusted to 7.0 ± 0.2 with 0.1 mol/L NaOH and centrifuged at 10000 rpm for 15 min. The supernatant was collected and freeze‐dried to obtain ORPH (Weijia et al. 2025).

ORPH was mixed with extraction solution (BTP‐KYP‐20250205‐09, Beijing Biotech Pack Scientific Co. Ltd), sonicated, passed through a 10 kDa ultrafiltration tube, and the sample was collected. The sample concentration was measured at A205 nm. After reduction, alkylation, and C18 desalting, the peptides were identified by Nano‐LC–MS/MS. The procedure is shown in Figure 1A. The specific methods are detailed in the Supplementary Information.

**FIGURE 1:**
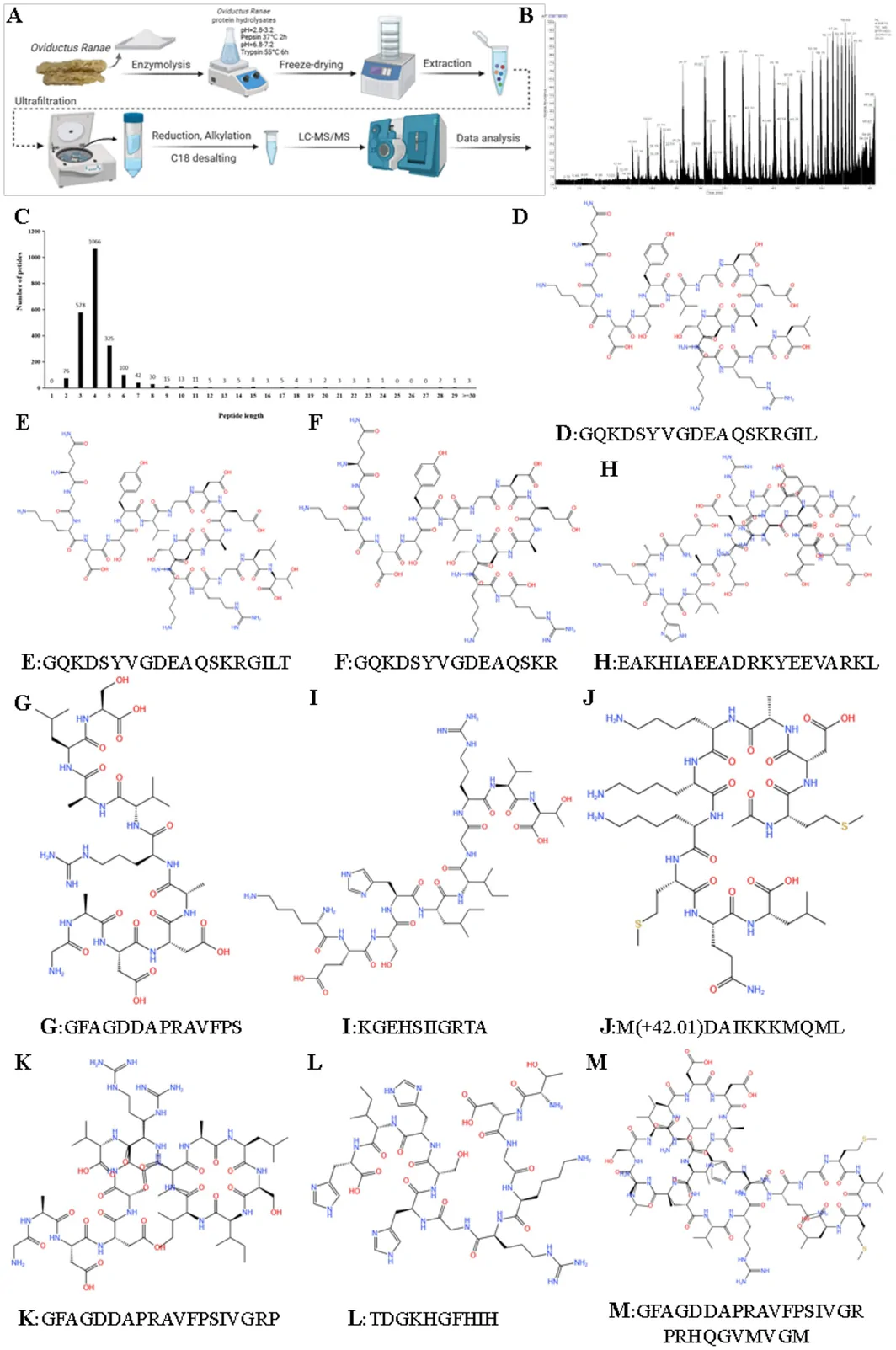
Isolation and Component Identification of ORPH. (A) Schematic of experimental design. (B) Total Ion Current (TIC) chromatogram. (C) Peptide length distribution of one sample. (D–M) Molecular formulas of the top ten peptides.

### 2.3 Animals and Experimental Design

60 SPF‐grade ICR mice, male, weighing 18–22 g, were purchased from Liaoning Changsheng Biotechnology Co. Ltd. (Jilin, China) and housed under standard conditions. The environmental temperature was maintained at 22°C ± 2°C, humidity at 50% ± 10%, and a 12‐h light/dark cycle was maintained, with food and water available ad libitum. Throughout the study, all animal handling and experiments were conducted in accordance with the recommendations of the National Standard of the People's Republic of China, ‘Guidelines for the Ethical Review of Laboratory Animal Welfare’ (GB/T 35892‐2018). The Animal Ethics Committee of Changchun University of Chinese Medicine approved all procedures involving animal experiments. After 1 week of acclimatization, the mice were randomly assigned to the following groups: Control, Model, Fluoxetine (3 mg/kg), high‐dose ORPH group (ORPH 0.8 g/kg), medium‐dose ORPH group (ORPH 0.4 g/kg), and low‐dose ORPH group (ORPH 0.2 g/kg), with 10 animals per group. Except for the Control group, a depression mouse model was established using CUMS. All stressors were initiated from the first week, with adjacent stressors differing from each other, and the stress lasted for 42 days. Drug treatment began on day 28. The Control and Model groups were administered an equal volume of physiological saline via oral gavage, while the remaining groups received the corresponding drugs, with treatment lasting for 14 days. The specific procedure is shown in Figure 2A. Body weight measurements and sucrose preference tests (SPT) were conducted weekly. Behavioral experiments were conducted after week 6. Following behavioral experiments, mouse feces were collected in a sterile environment, and mice were euthanized under anesthesia. Blood samples were collected from the orbital sinus via centrifugation to separate serum, and brain tissue was extracted, rapidly frozen in liquid nitrogen, or preserved in 4% paraformaldehyde for further biochemical analysis.

**FIGURE 2:**
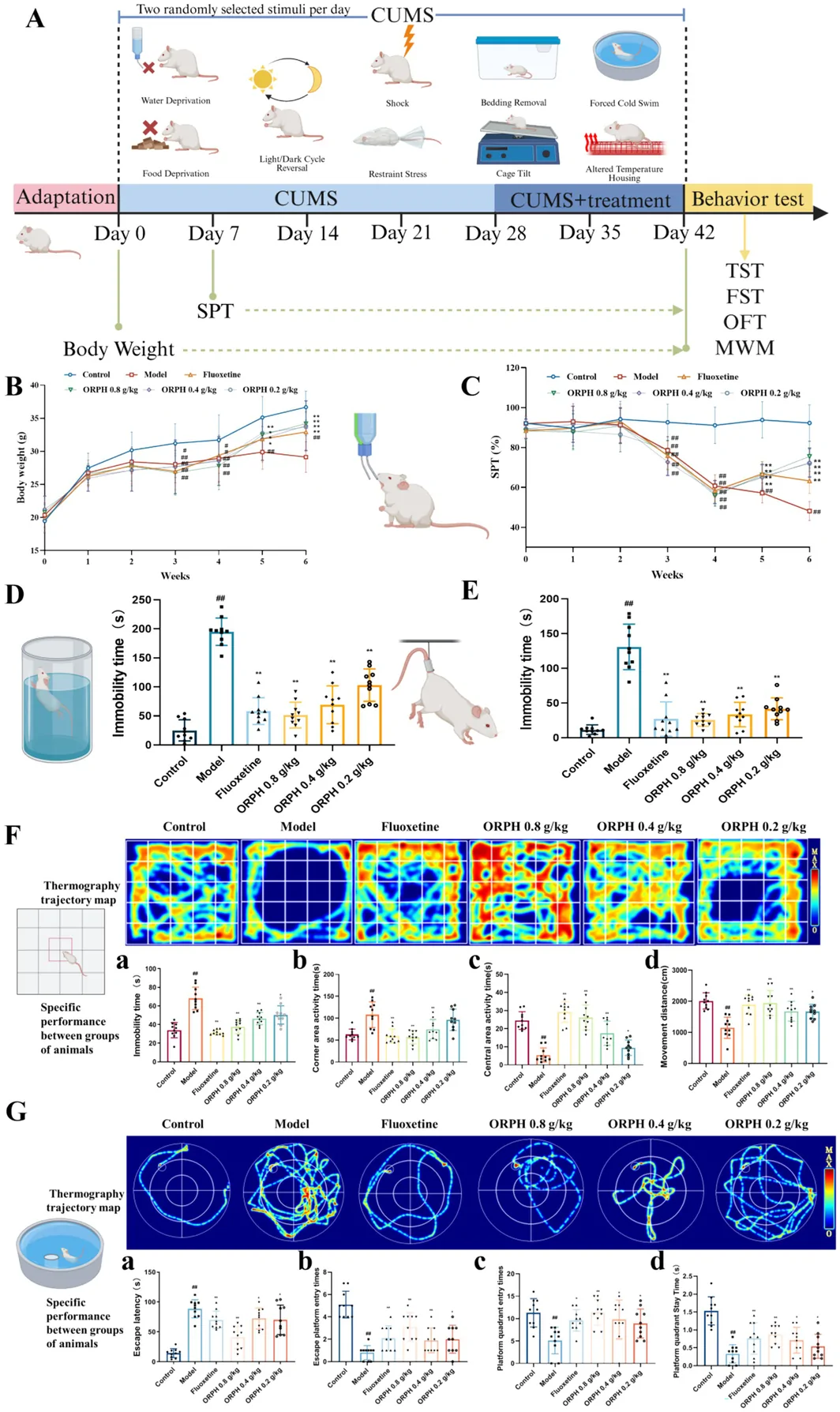
Effects of ORPH on behavioral outcomes in depressed mice. (A) Schematic of experimental design. (B) Body weight. (C) Sucrose preference ratio. (D) Immobility time in the FST. (E) Immobility time in the TST. (F) OFT locomotor trajectory plot: (a) immobility time, (b) corner activity time, (c) central activity time, (d) locomotor distance. (G) MWMT movement trajectory diagram: (a) escape latency; (b) entries into escape platform location; (c) entries into platform quadrant; (d) dwell time in platform quadrant. # and ## denote *p* < 0.05 and 0.01 compared with the Control group, respectively; * and ** denote *p* < 0.05 and 0.01 compared with the Model group, respectively.

### 2.4 Behavioral Tests

#### 2.4.1 Sucrose Preference Tests (SPT)

The SPT is an important behavioral test for assessing hedonic deficiency in mice. The test is conducted once a week, with the results from week 0 serving as the baseline. In the experiment, mice are given two identical water bottles (one containing distilled water and the other containing a 2% sucrose solution) for 24 h, and the sucrose preference rate of the mice is measured and recorded over the 24 h. Sucrose preference rate = sucrose water consumption/(distilled water consumption + sucrose water consumption) * 100% (Verharen et al. 2023).

#### 2.4.2 Forced Swimming Test (FST)

The FST is an experiment used to assess despair behavior in mice. Mice are placed in a cylindrical tank (diameter 10 cm, height 30 cm) filled with water to a depth of 15 cm (24°C ± 1°C). The time during which the mouse remains motionless for 5 min is measured. A mouse is considered motionless if there is no movement in its legs (Yankelevitch‐Yahav et al. 2015).

#### 2.4.3 Tail Suspension Test (TST)

The TST is an experiment to assess despair behavior in mice. The tip of the mouse's tail (1–2 cm) is secured to a horizontal bar with tape, with the head facing downward and the tail suspended 15–20 cm above the ground. The experiment lasts for 6 min, and the time the mouse remains motionless for the last 4 min is recorded. The mouse is completely passively suspended, and only occasional, slight limb movements are considered motionless (Maes et al. 1993).

#### 2.4.4 Open Field Test (OFT)

The OFT is used to assess the anxiety level and spontaneous motor activity of mice. Mice are placed in a square black box measuring 50 × 50 × 40 cm. After a 10‐min adaptation period, the mouse's movement trajectory is observed for 5 min using video tracking software Viewer 3.0 (Taimeng, China). The following parameters are calculated: movement distance (cm), immobility time (s), central area activity time (s), and corner area activity time (s). After each experiment, the test chamber is cleaned with 75% ethanol (Benrick et al. 2009).

#### 2.4.5 Morris Water Maze Test (MWMT)

The MWMT is used to assess the learning and memory abilities of mice. The experiment consists of two phases: a training phase and an experimental phase. Warm water is added to a circular pool (diameter 100 cm), with the water temperature controlled at (23 ± 1)°C. An escape platform (diameter 10 cm) is placed in the second quadrant. Mice are individually placed into the uncoloured water tank from different quadrants, and the time taken for them to reach the escape platform is observed. During the training period, if a mouse fails to reach the escape platform within 120 s, it is guided onto the platform. The training period lasted for 5 days. On the sixth day, the formal experiment was conducted. The pool was dyed opaque white using titanium dioxide. The WMT‐200 Morris Water Maze Analysis System (Taimeng, China) was used to place the mice in the pool for a spatial navigation experiment, and the escape latency of the mice was calculated. On the seventh day, a spatial exploration experiment was conducted to observe the number of times the mouse passed through the platform location within 120 s, the number of entries into the platform quadrant, and the time of stay in the platform quadrant (Bromley‐Brits et al. 2011).

### 2.5 Enzyme‐Linked Immunosorbent Assay (ELISA) Analysis

Quantitative analysis of corticosterone (CORT), 5‐Hydroxytryptamine (5‐HT), Norepinephrine (NE), Interleukin‐6 (IL‐6), Tumor Necrosis Factor‐α (TNF‐α), and Interleukin‐1β (IL‐1β) levels in mouse brain tissue, as well as Adrenocorticotropic Hormone (ACTH), Corticotropin‐Releasing Hormone (CRH), IL‐6, TNF‐α, and IL‐1β levels in serum. All procedures were strictly conducted in accordance with the instructions provided in the ELISA kit. The ELISA kits for CORT (MM‐0061M2), ACTH (MM‐0554M2), CRH (MM‐0509M2), 5‐HT (MM‐0443M2), NE (MM‐0876M2), IL‐6 (MM‐0163M2), IL‐1β (MM‐0040M2), TNF‐α (MM‐0132M2), Trp (MM‐926267O1), Kyn (MM‐928072O1), Cer (MM‐927708O1), and S1P (MM‐91866O1) were purchased from Jiangsu Enzyme Immunoassay. The detailed parameters are shown in Table S1. An enzyme‐linked immunosorbent assay reader (SUNERGY‐HTX, USA) was used to record the OD values of the samples, and a standard curve was plotted to calculate the concentrations.

### 2.6 H&E Staining

Mouse brain tissue was preserved in 4% paraformaldehyde, dehydrated, sealed with wax prior to embedding, and cooled on a −20°C freezing platform. Paraffin‐embedded tissue sections were approximately 4 μm thick. The sections were floated in a water bath at 40°C to flatten the tissue, mounted on slides, and baked at 60°C. The sections were dewaxed in water, stained with Harris's hematoxylin, differentiated with 1% hydrochloric acid alcohol, counterstained with 0.6% ammonia solution, rinsed, and stained with eosin solution. After dehydration, the sections were mounted with neutral binder. Observations were made under an optical microscope (Nikon, Japan), and images were collected for analysis.

### 2.7 Tunel Staining

Embed the paraffin‐embedded sections in wax, then dewax them in water. Digest with proteinase K at 37°C for 30 min, then wash with PBS. Treat with membrane disruption solution at room temperature for 20 min, then wash with PBS. Add the TUNEL reaction solution and incubate in a water bath at 37°C for 2 h, then wash with PBS. Stain the nuclei with DAPI for 10 min, mount the slides with anti‐fluorescence quenching, and observe and collect images under a fluorescence microscope (Nikon, Japan).

### 2.8 16S rRNA Sequencing

Prior to euthanizing the mice, six mice were randomly selected from each group. The contents of the caecum were collected and placed into sterile centrifuge tubes, rapidly frozen in liquid nitrogen, and stored at −80°C to prevent DNA degradation. DNA was extracted using the TGuide S96 magnetic bead method according to the manufacturer's instructions. PCR amplification of the V3 + V4 variable regions was performed using region‐specific primers. The PCR amplification primer sequences were 5′‐ACTCCTACGGGAGGCAGCA‐3′ and 5′‐GGACTACHVGGGTWTCTAAT‐3′. Data analysis was conducted after sequencing. The analysis process used USEARCH (version 10.0) to cluster sequences at a similarity level of 97%, with a default threshold of 0.005% of the total number of sequenced sequences to filter OTUs. QIIME2 (version 2020.6) was used to perform noise reduction on the quality‐controlled data using the DADA2 method. The default threshold for filtering ASVs was set at 0.005% of the total number of sequenced sequences. The representative sequences of ASVs were aligned against the GREENGENES database to obtain the species classification information table. Finally, predictive analyses were conducted on the samples, including α‐diversity indices, β‐diversity indices, and microbial community composition at the feature sequence level.

### 2.9 Untargeted Metabolomics Analysis

Non‐targeted analysis was performed using LC–MS equipped with an electrospray ionization (ESI) source (Biomarker, Beijing, China). Liquid chromatography separation was carried out using a UPLC system equipped with an Acquity UPLC HSS T3 column (2.1 mm × 100 mm, 1.8 μm). Specific parameters: Mobile phase A was 0.1% formic acid aqueous solution, mobile phase B was 0.1% formic acid acetonitrile solution, gradient elution (0–0.25 min, 98%–2% B; 0.25–10.0 min, 2%–98% B; 10.0–13.0 min, 2%–98% B; 13.0–15.0 min, 98%–2% B), flow rate 400 μL·min^−1^, injection Volume 1 μL. Mass spectrometry parameters were set as follows: positive and negative ion modes were scanned, low collision energy 2 V, high collision energy range 10–40 V, cone voltage 30 V, ion source temperature 150°C, desolvation gas temperature 500°C, backflush gas flow rate 50 L·h^−1^, desolvation gas flow rate 800 L·h^−1^, capillary voltage 2 kV (ESI^+^), −1.5 kV (ESI^−^), and mass range m/z 50–1200. In the MassLynx V4.2 data acquisition software, dual‐channel data acquisition is performed simultaneously for both low and high collision energies in each data acquisition cycle. The peak areas detected by the mass spectrometer are normalized using the library search software Progenesis QI 4.0 for analysis.

### 2.10 Pathway Blockade Verification Experiments

36 SPF male ICR mice, weighing 18–22 g, were purchased from Liaoning Changsheng Biotechnology Co. Ltd. (Jilin, China). The feeding environment was the same as previously described. After 1 week of acclimatization, the mice were randomly divided into Control, Model, ORPH, ABX, PCPA, and SKI‐II groups, with 6 mice in each group. Except for the Control group, a depression mouse model was established using CUMS. All stress stimuli started from the first week, with no two consecutive stimuli being the same, and the stimulation lasted for 42 days. In the ABX group, a broad‐spectrum antibiotic mixture (1 g/L ampicillin sodium salt (BS923), 1 g/L metronidazole (M109874), 0.5 g/L neomycin sulfate (N109017), and 0.5 g/L vancomycin hydrochloride (V105495)) was added to the drinking water from day 21 to day 28 to deplete the gut microbiota. Drug administration began on day 28. The Control and Model groups received an equal volume of normal saline by gavage. All other groups were administered 0.8 g/kg ORPH. The PCPA group received an intraperitoneal injection of 250 mg/kg PCPA (HY‐B1368) (Foltran et al. 2020), and the SKI‐II group received an intraperitoneal injection of 50 mg/kg SKI‐II (HY‐13822) (Chiba et al. 2010). The detailed procedure is shown in Figure 11A. The SPT was conducted every 2 weeks. After the 6th week, behavioral experiments were performed. Following the behavioral tests, the mice were euthanized under anesthesia. Blood samples were collected from the retro‐orbital sinus and centrifuged to separate serum. Brain tissues were then harvested, rapidly frozen in liquid nitrogen, and stored for subsequent biochemical assays.

### 2.11 Statistical Analysis

Analysis was performed using SPSS software, and results are expressed as mean ± standard deviation (SD). Graphs were performed using GraphPad Prism software. A mixed‐effects model was applied for repeated‐measures behavioral data collected at multiple time points. Separate linear mixed‐effects models were fitted for body weight and sucrose preference rate, each including week, group, and their interaction as fixed effects, with mouse as a random intercept. The fixed‐effect test results are summarized in Table S2. One‐way analysis of variance (ANOVA) was performed to assess the significance of differences between means. Tukey's HSD was used for correction. *p* < 0.05 was considered statistically significant.

## 3 Results

### 3.1 Composition Analysis of ORPH

LC–MS/MS was applied for further separation and identification of the components in ORPH (Figure 1B). A total of 2308 peptides were identified, which were mainly distributed in the molecular weight range of 2–6 kDa (Figure 1C). Based on the software scoring ranking, the structures of the top ten peptides were drawn using Kingdraw (Figure 1D–M). Table S3 summarizes the protein accession numbers, amino acid sequences, matching scores and chromatographic peak areas of the top 10 peptides ranked by identification score. Detailed information of each peptide is provided in the Table S3.

### 3.2 Effects of ORPH on Body Weight and Behavior in Depressed Mice

To evaluate ORPH's impact on depressed mice, we employed the CUMS model. As illustrated in Figure 2A, fluctuations in body weight and sucrose preference rate were monitored throughout the feeding period. Twenty‐one days post‐CUMS modeling, body weights in all groups were significantly reduced compared to the Control group, with ORPH treatment gradually restoring weights to normal levels (Figure 2B). From day 21 onwards, the sucrose preference rate decreased significantly across each group. By day 28, the sucrose preference rate had fallen from baseline to 55.80%, indicating pronounced anhedonia in each group. Following ORPH intervention, this rate rebounded to 73.32% (Figure 2C). At the conclusion of treatment, despair behavior, anxiety, spontaneous activity, and learning/memory capacity were assessed via FST, TST, OFT, and MWMT. In FST and TST, Model mice exhibited significantly prolonged immobility periods, which decreased to varying degrees post‐treatment (Figure 2D,E). In the OFT, Model mice exhibited markedly increased immobility and corner exploration times alongside reduced central zone activity and total locomotor distance, with treatment inducing a reversal trend (Figure 2F). The MWMT revealed significantly prolonged escape latency in depressive model mice, alongside reduced platform crossings within 120 s, platform quadrant entries, and quadrant dwell time, all of which improved following treatment (Figure 2G). During the place navigation training phase, escape latency gradually decreased with training days; the detailed results are shown in Figure S1. Results indicate that all dose groups of Fluoxetine and ORPH significantly ameliorated depressive‐like behavior in the model mice. Notably, ORPH exhibited a pronounced dose‐dependent effect, with behavioral improvement positively correlated to administered dose.

### 3.3 Effects of ORPH on Biochemical Markers in Depressed Mice

To further elucidate the mechanism of action of ORPH, we assessed levels of inflammatory cytokines, HPA axis‐related hormones, and neurotransmitters. CUMS induces neuroinflammation in the mouse brain. As hypothesized, pro‐inflammatory factors (IL‐6, IL‐1β, and TNF‐α) were significantly elevated in both serum and brain tissue of the Model group (Figure 3A–C,F–H). Concurrently, excessive HPA axis activation resulted in markedly increased serum ACTH and CRH levels (Figure 3D,E), while CORT levels in brain tissue were markedly elevated (Figure 3I). Conversely, 5‐HT and NE concentrations were significantly reduced (Figure 3J,K). Treatment with the compound significantly reversed these alterations. We hypothesize that ORPH may indirectly attenuate central inflammatory responses by suppressing peripheral inflammation, such as through reduced pro‐inflammatory cytokine release.

**FIGURE 3:**
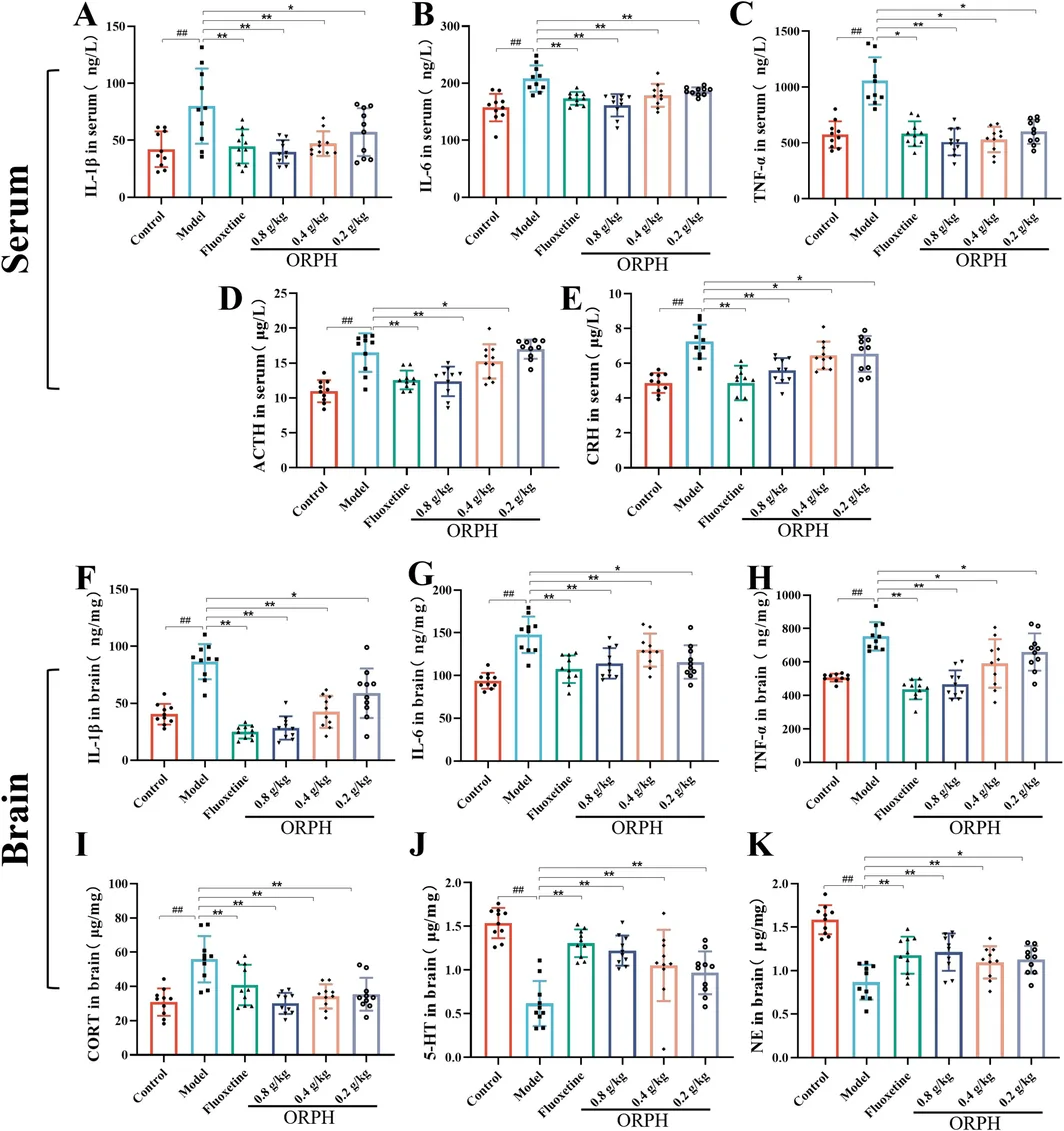
Effects of ORPH on biochemical parameters in serum and brain tissue of depressed mice. (A) Serum IL‐1β levels. (B) Serum IL‐6 levels. (C) Serum TNF‐α levels. (D) Serum ACTH levels. (E) Serum CRH levels. (F) Brain tissue IL‐1β levels. (G) IL‐6 levels in brain tissue. (H) TNF‐α levels in brain tissue. (I) CORT levels in brain tissue. (J) 5‐HT levels in brain tissue. (K) NE levels in brain tissue. # and ## denote *p* < 0.05 and 0.01 compared with the Control group, respectively; * and ** denote *p* < 0.05 and 0.01 compared with the Model group, respectively.

### 3.4 ORPH Repairs Histopathological Damage in the Hippocampal Tissue of Depressed Mice

Depression exhibits multidimensional pathophysiological associations with hippocampal tissue. This study employed H&E staining to analyze damage in the CA1, CA3, and DG regions of the hippocampal tissue from depressed mice. Results demonstrated that in the Control group, cells within the hippocampal CA1, CA3, and DG regions exhibited uniform size and orderly arrangement, with no evidence of cell necrosis or hypertrophy. In the Model group, the number of cells in the hippocampal CA1 region was markedly reduced, accompanied by nuclear condensation and necrosis. In the CA3 and DG regions, disordered cell arrangement, widened intercellular spaces, and morphological alterations were observable. Compared with the Model group, all treatment groups exhibited increased cell numbers in the CA1, CA3, and DG regions, with more regular cell morphology, orderly intercellular arrangement, clear boundaries, and reduced damage (Figure 4). In mouse models of depression, apoptosis constitutes a key pathological feature, particularly evident in emotion‐regulating brain regions such as the hippocampus. Therefore, we assessed hippocampal CA1 apoptosis levels via TUNEL staining. The Model group exhibited markedly increased apoptotic cells in hippocampal tissue. Following treatment, apoptotic cell numbers decreased significantly in all drug‐treated groups (Figure 5).

**FIGURE 4:**
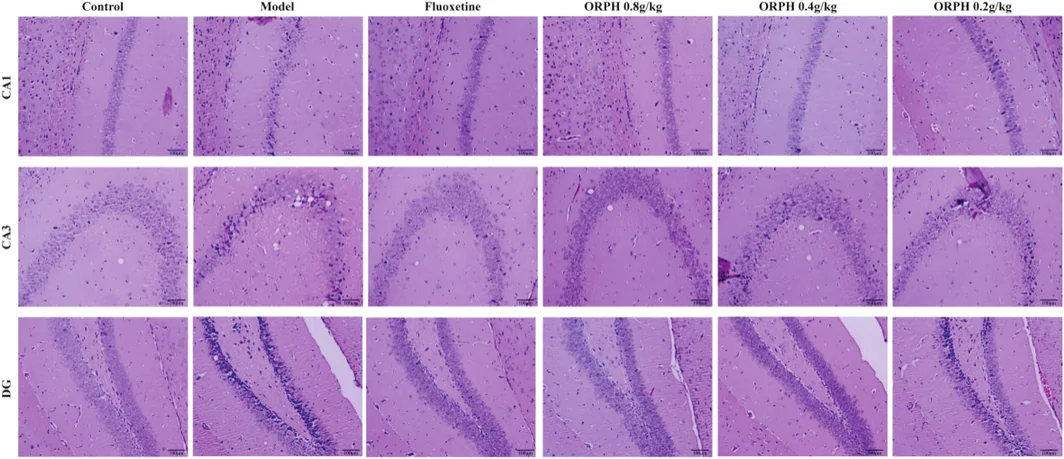
ORPH ameliorates hippocampal damage in depressed mice (×200, 100 μm).

**FIGURE 5:**
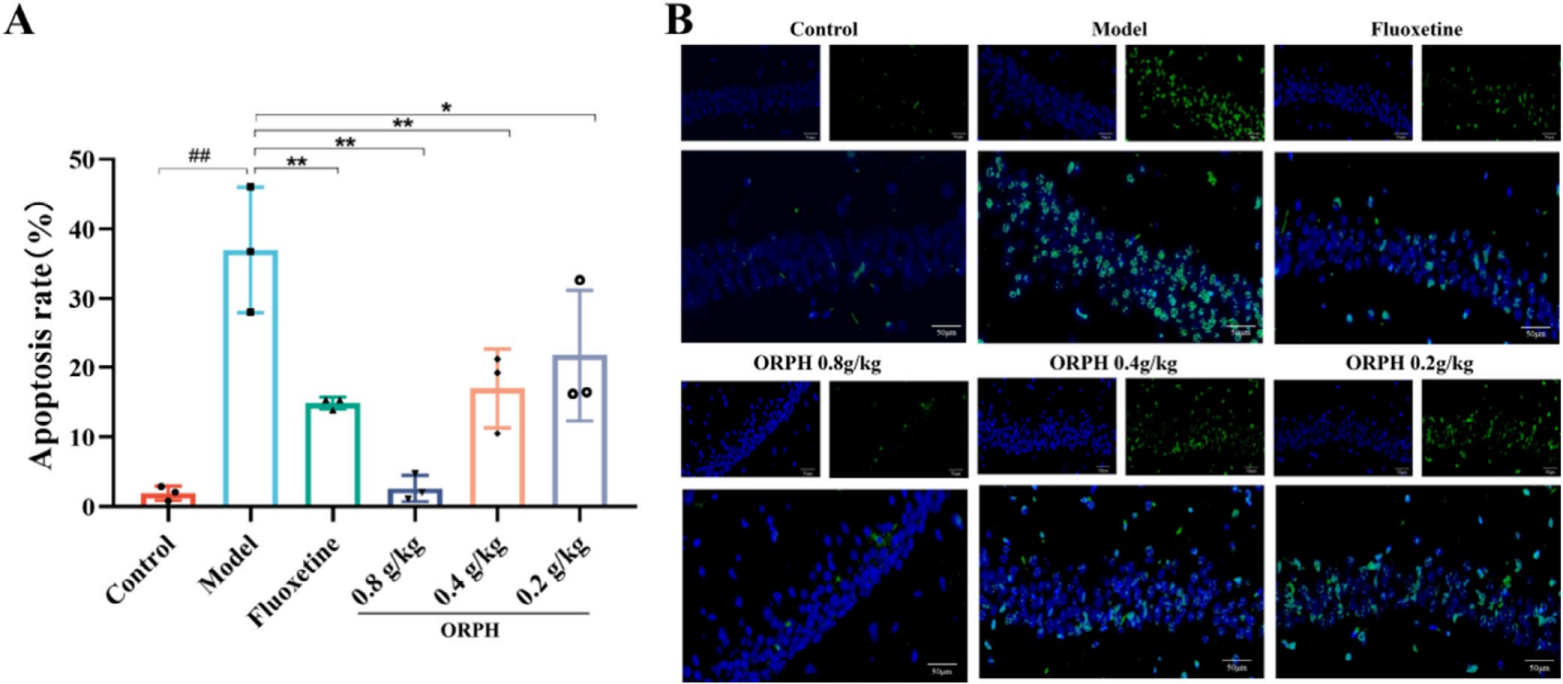
ORPH ameliorates hippocampal tissue apoptosis levels in depressed mice (×200, 50 μm) (A) Apoptosis rate. (B) TUNEL staining. (Green: Apoptotic cells; DAPI: Cell nuclei). ## denote *p* < 0.01 compared with the Control group, respectively; * and ** denote *p* < 0.05 and 0.01 compared with the Model group, respectively.

### 3.5 Effects of ORPH on Brain Tissue Metabolites in Depressed Mice

This study randomly selected six mice each from the Control group, Model group, and ORPH 0.8 g/kg group. Brain tissue metabolites were analyzed using non‐targeted metabolomics technology, with the groups subsequently renamed as Control, Model, and ORPH groups. To assess the stability of non‐targeted metabolomics, principal component analysis was performed. Results revealed dispersed sample positions among the three groups, indicating significant alterations in the overall trends of brain tissue metabolites (Figure 6A). Subsequently, OPLS‐DA plots demonstrated distinct metabolic clusters among Control, Model, and ORPH groups (Figure 6B,C), and permutation testing was performed to validate the model, with the results shown in Figure S2. Differential metabolites were screened using a volcano plot, applying criteria of VIP > 1.0 and *p* < 0.05. Compared to the Control group, 444 differential metabolites were identified in the Model group brain tissue (Figure 6D), comprising 173 upregulated (red dots) and 271 downregulated (blue dots) metabolites. Furthermore, compared with the Model group, 77 upregulated (red dots) and 186 downregulated metabolites (blue dots) were detected in the ORPH group brain tissue (Figure 6E). The intersection of the two groups revealed 211 shared differentially expressed metabolites, 29 of which exhibited opposite trends (Table 1). Pathway enrichment analysis based on this data (Figure 6F) showed significant enrichment of biomarkers in Glycerophospholipid metabolism and Sphingolipid metabolism. Neuroinflammatory responses disrupt sphingolipid metabolism (Ayub et al. 2021), particularly affecting three key metabolites: Ganglioside GM3 (d18:1/9Z‐18:1), Galabiosylceramide (d18:1/9Z‐18:1), and N, N‐Dimethylsphingosine, which were significantly upregulated in the Model group and markedly downregulated following ORPH treatment (Figure 6G–I). Collectively, these findings indicate that ORPH treatment significantly alters the metabolic profile of brain tissue in CUMS mice.

**FIGURE 6:**
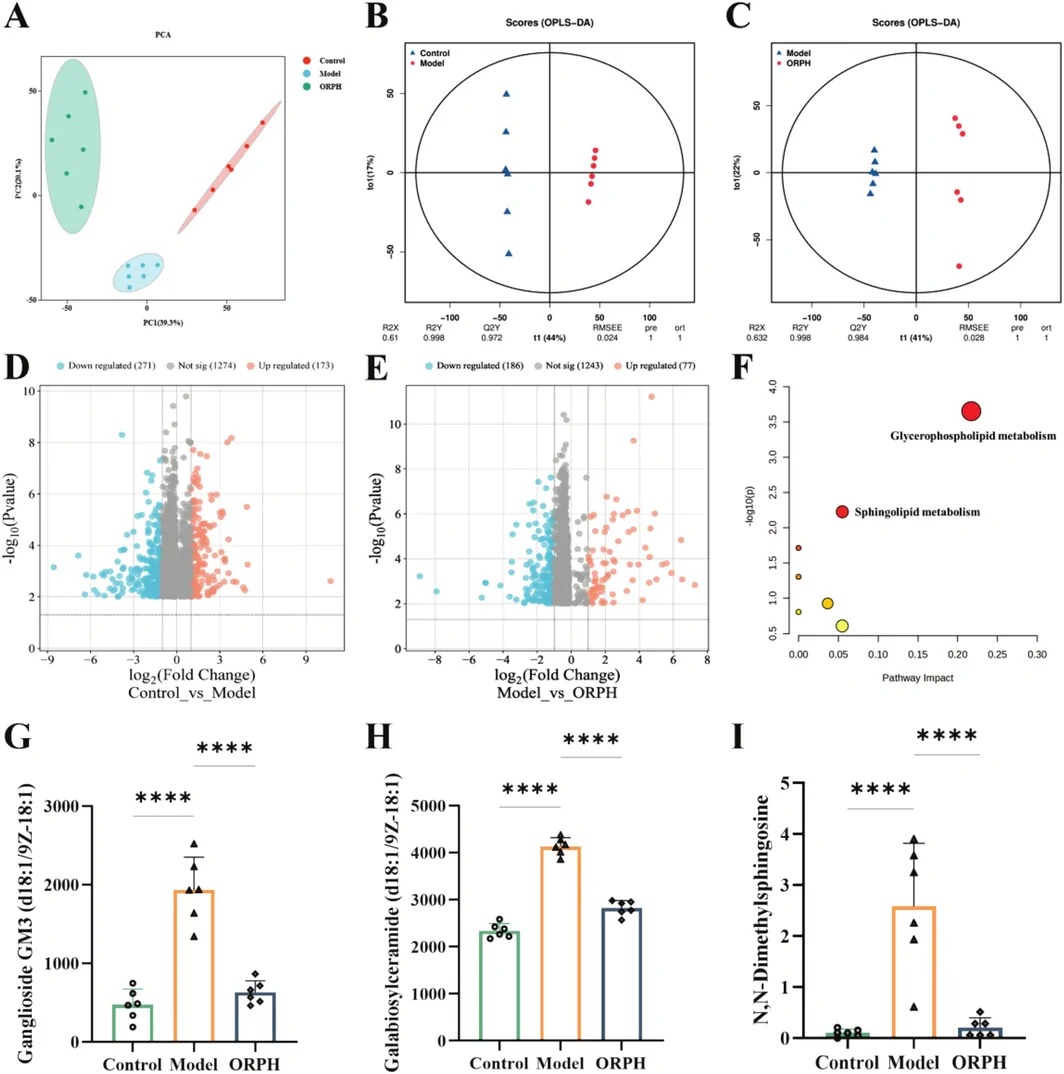
Alterations in brain tissue metabolites of depressed mice following ORPH treatment. (A) Principal Component Analysis (PCA). (B) OPLS‐DA analysis of Control versus Model groups, *R* ^*2*^ *Y* = 0.998, *Q* ^*2*^ = 0.972. (C) OPLS‐DA analysis of Model versus ORPH groups, *R* ^*2*^ *Y* = 0.996, *Q* ^*2*^ = 0.984. (D) Volcano plot of Control versus Model groups. (E) Volcano plot of Model versus ORPH groups. (F) KEGG pathway enrichment diagram. (G) Differential expression levels of ganglioside GM3 (d18:1/9Z‐18:1). (H) Differential expression levels of galabiosylceramide (d18:1/9Z‐18:1). (I) Differential expression levels of N, N‐dimethylsphingosine. # and ## denote *p* < 0.05 and 0.01 compared to the Control group, respectively; **** denote *p* < 0.0001 compared to the Model group.

**TABLE 1:** Effects of ORPH on biomarkers in brain tissue.

Name	Control_Mean	Model_Mean	ORPH_Mean	C‐M	M‐O	C‐M	M‐O
				Regulated	Regulated	FDR/q	FDR/q
Retinol/Retinol skeleton	585.76	1292.71	636.08	↑**	↓**	0.002	0.005
PE (16:0/22:5 (7Z,10Z,13Z,16Z,19Z))	51.70	154.26	58.58	↑**	↓**	0.028	0.009
PC (20:4 (5Z,8Z,11Z,14Z)/P‐18:1 (11Z))	1338.96	2398.86	1603.47	↑**	↓**	0.006	0.010
PC (20:4 (5Z,8Z,11Z,14Z)/22:6 (4Z,7Z,10Z,13Z,16Z,19Z))	7475.15	15747.81	12180.98	↑**	↓**	< 0.001	0.004
PC (20:3 (5Z,8Z,11Z)/22:6 (4Z,7Z,10Z,13Z,16Z,19Z))	272.97	430.00	352.78	↑**	↓**	< d0.001	0.003
Oleoylcarnitine	45.42	107.39	47.43	↑**	↓**	0.002	0.001
*N*,*N*‐Dimethylsphingosine	0.10	2.59	0.21	↑**	↓**	0.016	0.019
MG (0:0/20:1 (11Z)/0:0)	209.91	632.82	305.34	↑**	↓**	< 0.001	0.014
LysoPE (20:2 (11Z,14Z)/0:0)	26.16	66.33	35.42	↑**	↓**	0.002	0.012
LysoPE (0:0/20:5 (5Z,8Z,11Z,14Z,17Z))	594.34	841.32	606.30	↑**	↓**	0.009	0.010
LysoPE (0:0/20:2 (11Z,14Z))	926.60	1358.30	1001.47	↑**	↓**	< 0.001	0.002
LysoPE (0:0/20:1 (11Z))	1841.30	3882.56	2303.17	↑**	↓**	< 0.001	0.015
LysoPE (0:0/20:0)	66.24	122.85	75.32	↑**	↓**	0.002	0.001
LysoPC (22:6 (4Z,7Z,10Z,13Z,16Z,19Z))	138643.38	170944.58	140669.22	↑**	↓**	0.005	0.009
LysoPC (22:1 (13Z))	2.32	28.32	10.27	↑**	↓**	0.006	0.030
LysoPC (18:2 (9Z,12Z))	3.67	24.36	6.34	↑**	↓**	0.016	0.026
Lithocholic acid glycine conjugate	152.29	200.24	164.00	↑**	↓**	0.014	0.003
Indoleacrylic acid	60.82	0.73	19.18	↓**	↑**	0.025	< 0.001
Ganglioside GM3 (d18:1/9Z‐18:1)	475.40	1933.76	632.51	↑**	↓**	0.001	0.002
Galabiosylceramide (d18:1/9Z‐18:1)	2341.25	4132.98	2823.40	↑**	↓**	< 0.001	< 0.001
Dodecanedioylcarnitine	11.50	27.03	14.10	↑**	↓**	0.001	< 0.001
DHAP (18:0)	434.63	819.86	453.64	↑**	↓**	< 0.001	< 0.001
DG (15:0/22:6 (4Z,7Z,10Z,13Z,16Z,19Z)/0:0)	30.76	45.35	31.57	↑**	↓**	0.003	0.011
6‐Ketoestriol	40.29	114.39	74.08	↑**	↓**	< 0.001	0.009
5‐Phosphoribosyl‐4‐carboxy‐5‐aminoimidazole	394.57	699.02	425.35	↑**	↓**	0.002	0.012
4‐OH‐Retinal	44.45	67.64	49.09	↑**	↓**	0.005	< 0.001
1b‐Hydroxycholic acid	15.72	50.56	20.99	↑**	↓**	< 0.001	< 0.001
12,15‐epoxy‐13‐methyleicosa‐12,14‐dienoic acid	116.67	214.87	136.49	↑**	↓**	0.003	0.008
11‐Hydroxyeicosatetraenoate glyceryl ester	144.80	190.20	150.27	↑**	↓**	0.003	< 0.001

** denote *p* < 0.01.

### 3.6 ORPH Ameliorates Gut Microbiota Dysbiosis in Depressed Mice

We employed 16S rRNA sequencing to analyze colonic fecal samples from mice. Analysis of α diversity revealed significant increases in the Ace index and Chao1 index following ORPH treatment, whilst the Shannon index and Simpson index showed no significant alterations (Figure 7A–D). This suggests ORPH modifies species abundance within the gut community structure without markedly affecting species diversity. Subsequently, β‐diversity analyses including PLS‐DA, PcoA, and NMDS revealed significant separation of gut microbial β‐diversity among groups (Figure 7E–G). To further investigate ORPH's effects on the gut microbiota of depressed mice, we analyzed microbial composition at the phylum and genus levels across the three groups. Across all taxonomic groups, *Bacteroidota* and *Bacillota* were the dominant phyla. CUMS‐induced depressive‐like behavior exerted varying degrees of influence on the gut microbiota of mice (Figure 7I,J). For instance, following CUMS stimulation, the Model group exhibited reduced *Bacteroidota*, increased *Bacillota*, and elevated *Bacteroidota/Bacillota* ratios (Figure 7H). Previous studies have reported changes in *Bacillota* and *Bacteroidota* abundance in depression models (Zheng et al. 2020). Through investigation of key microbial communities involving *Bacillota, Pseudomonadota, Bacteroidota, Actinobacteria*, and *Verrucomicrobia* (Figure 8), we found that following ORPH treatment, the relative abundance of *Bacillota* and *Pseudomonadota* was significantly reduced in depressed mice. In contrast, the abundance of *Bacteroidota, Actinobacteria*, and *Verrucomicrobia* was markedly increased. Specifically, taxa reported to possess pro‐inflammatory potential such as *Clostridium, Enterococcus, Escherichia‐Shigella*, and *Desulfovibrio* exhibited markedly diminished abundance. In contrast, taxa with putative anti‐inflammatory and SCFA‐producing capacities, including *Lactobacillus, Bifidobacterium*, and *Akkermansia*, showed markedly increased abundance.

**FIGURE 7:**
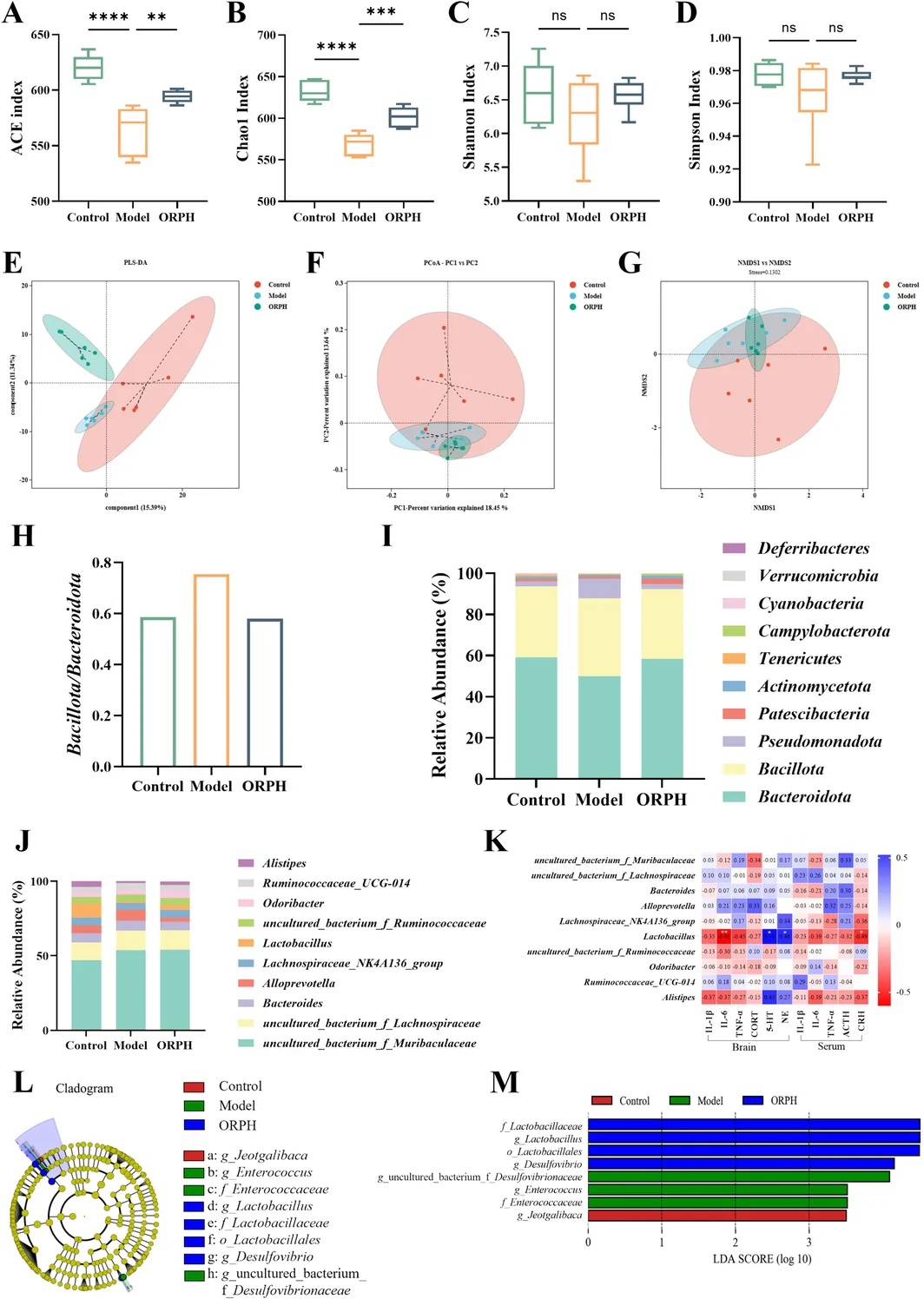
Effects of ORPH on the gut microbiota of depressed mice. (A–D) α‐diversity analysis. (E–G) β‐diversity analysis. (H) *Bacillota*/*Bacteroidota* ratio. (I) Phylum‐level microbial composition results. (J) Genus‐level microbial composition results. (K) Correlation analysis. (L–M) LefSe analysis. **, and **** denote *p* < 0.01, and 0.0001 respectively compared with the Model group.

**FIGURE 8:**
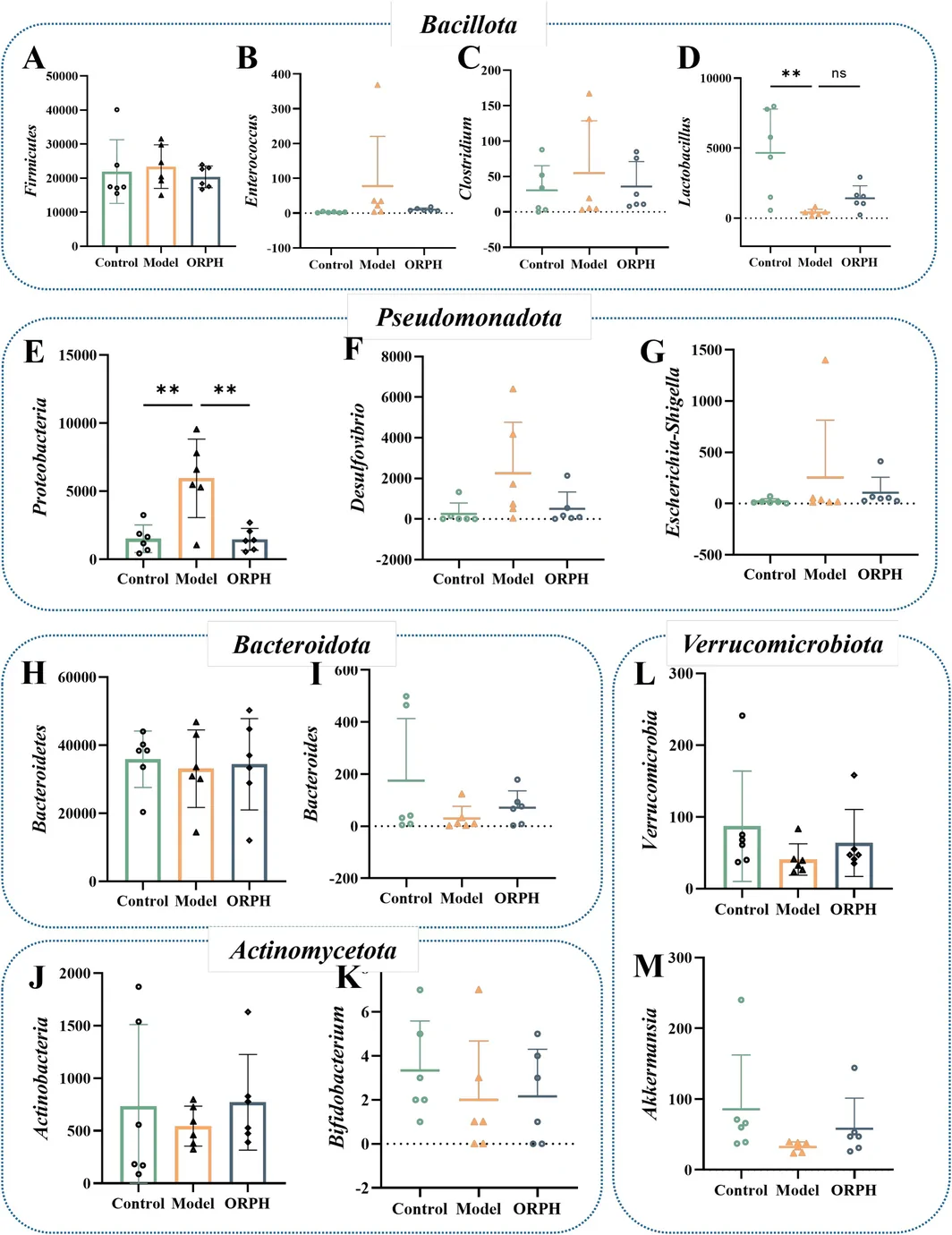
Effects of ORPH on key gut microbiota in depressed mice. (A–D) *Bacillota*. (E–G) *Pseudomonadota*. (H–I) *Bacteroidota*. (J–K) *Actinomycetota*. (L–M) *Verrucomicrobiota*. ** denote *p* < 0.01, compared with the Model group.

At the genus level, *Lactobacillus* abundance was significantly reduced in the Model group mice. To further identify the dominant microbial communities in each group, LefSe analysis (Figure 7L,M) revealed that the dominant community in the Control group was genus *Jeotgalibaca*, while the Model group was dominated by uncultured_bacterium_*Desulfovibrionacea*e and *Enterococcus* (family *Enterococcaceae*). The ORPH group exhibited dominant communities of genus *Desulfovibrio, Lactobacillales* (order), *Lactobacillaceae* (family), and *Lactobacillus* (genus). Research indicates that inflammatory responses cause gut barrier damage, leading to overgrowth of *Enterococcus* (Zitvogel and Kroemer 2019). The abundance of *Enterococcus*, a pro‐inflammatory taxon, was significantly reduced upon ORPH treatment (Figure 8B). *Desulfovibrionaceae* exhibited marked elevation in depression patients. Although residual levels persisted post‐ORPH treatment, their abundance remained markedly lower than in the Model group (Figure 8F). *Lactobacillus*, a genus widely reported to produce SCFAs, has been shown to effectively reduce inflammation levels when supplemented (Li et al. 2022), this raises the possibility that ORPH may modulate SCFA availability. Pearson correlation analysis between biochemical markers and Genus revealed (Figure 7K, Figure S3) that *Lactobacillus* exhibited a negative correlation trend with pro‐inflammatory factors (IL‐6, IL‐1β, TNF‐α) and a positive correlation trend with 5‐HT and NE levels. This further suggests that ORPH may prevent depression by regulating the relative abundance of *Lactobacillus*.

### 3.7 Effects of ORPH on Fecal Metabolites in Depressed Mice

To further investigate, we employed non‐targeted metabolomics to analyze fecal metabolites. Principal Component Analysis (PCA) revealed a clustering tendency within the three fecal sample groups, while inter‐group samples exhibited dispersion, indicating a significant shift in overall metabolic patterns (Figure 9A). Subsequent pairwise PCA analysis revealed significant separation between the Control and Model group fecal tissue metabolites (Figure 9B), while the Model group exhibited significant separation from the ORPH group (Figure 9C). Volcanic plots were employed to visually screen differential metabolites, using VIP values > 1.0 and *p* < 0.05 as screening criteria. Compared to the Control group, 1077 differential metabolites were identified in the fecal tissue of the Model group (Figure 9D), comprising 619 upregulated metabolites (red dots) and 458 downregulated metabolites (blue dots). Additionally, compared to the Model group, 365 upregulated (red dots) and 366 downregulated metabolites (blue dots) were detected in the ORPH group fecal tissue (Figure 9E). The intersection of these two groups revealed 231 shared differentially expressed metabolites, 33 of which exhibited opposite trends (Table 2). Pathway enrichment analysis based on this data (Figure 9F) identified significantly associated pathways, including Purine metabolism, Sphingolipid metabolism, and Tryptophan metabolism. Further statistical analysis of key metabolites revealed that compared to the Control group, Phytosphingosine 1‐phosphate expression was significantly downregulated in the Model group but significantly upregulated following ORPH treatment (Figure 10B). Conversely, 3‐Dehydrosphinganine and NPC expression were significantly upregulated in the Model group but significantly downregulated after ORPH treatment (Figure 10A,C). We generated a heatmap (Figure 10D) to further illustrate the differences in metabolites among the groups. Compared with the Control group, Indole‐3‐ethanol (Figure 10E) was significantly downregulated in the Model group, while 7,8‐dihydro‐7,8‐dihydroxykynurenate, 2‐aminobenzoic acid, and 2‐amino‐3‐carboxymuconic acid semialdehyde were significantly upregulated in the Model group, with both showing marked recovery following ORPH treatment (Figure 10F–H). These alterations suggest that ORPH modulates fecal sphingolipid metabolic dysregulation and inhibits tryptophan metabolic diversion.

**FIGURE 9:**
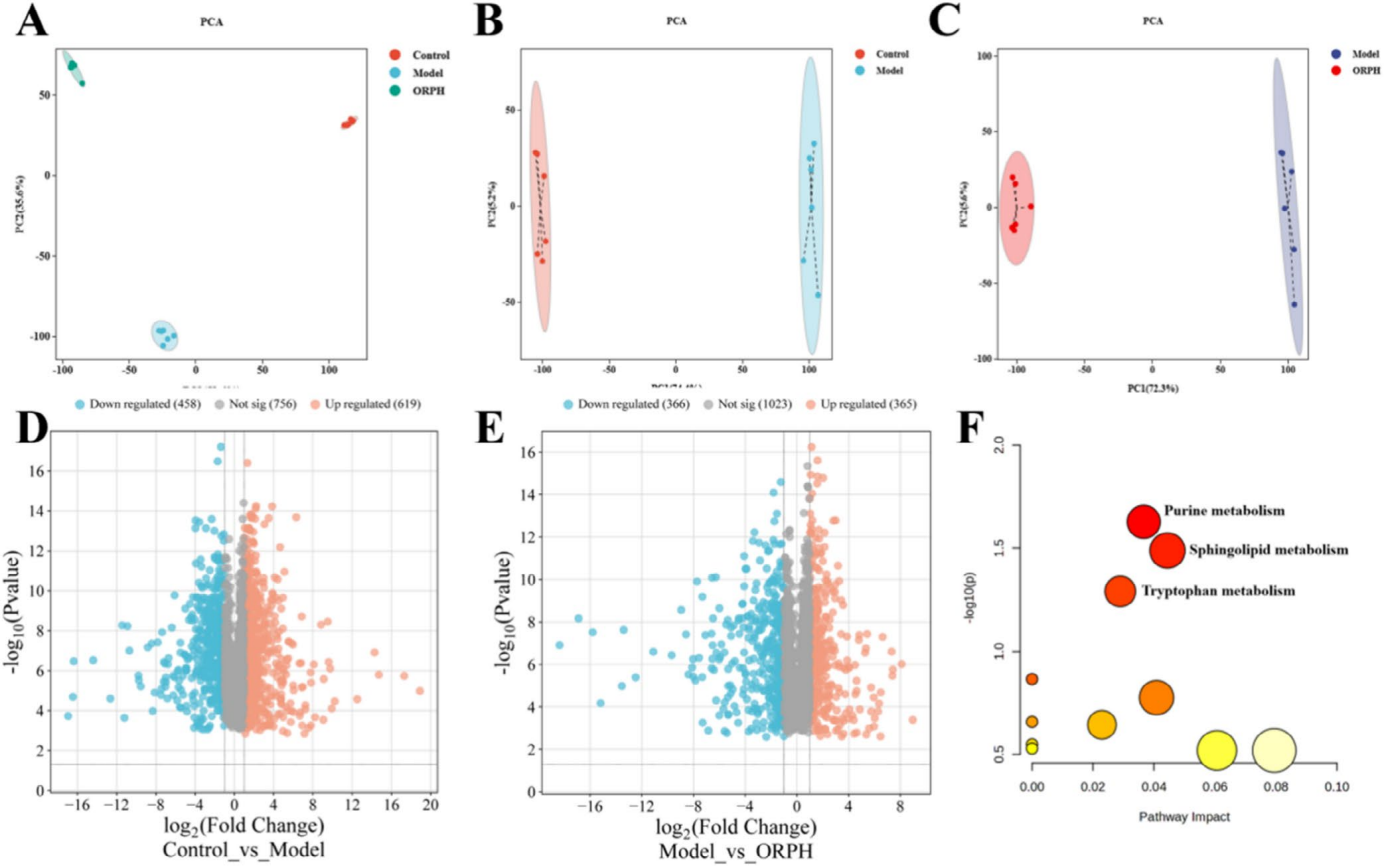
Alterations in fecal metabolites of depressed mice following ORPH treatment. (A–C) PCA. (D) Volcano plot for Control/Model group. (E) Volcano plot for Model/ORPH group. (F) KEGG pathway enrichment diagram.

**TABLE 2:** Effects of ORPH on biomarkers in fecal tissue.

Name	Control_Mean	Model_Mean	ORPH_Mean	C‐M	M‐O	C‐M	M‐O
				Regulated	Regulated	FDR/q	FDR/q
Xanthosine	121.21	598.21	190.83	↑**	↓**	< 0.001	< 0.001
THF‐L‐glutamate	7654.42	51.80	234.90	↓**	↑**	< 0.001	< 0.001
PS (14:0/16:1 (9Z))	1301.42	25.22	130.33	↓**	↑**	< 0.001	< 0.001
PI (10:0/16:0)	7121.71	95.32	539.86	↓**	↑**	< 0.001	< 0.001
Phytosphingosine 1‐phosphate	2738.47	175.02	961.68	↓**	↑**	< 0.001	< 0.001
PGD3	17141.97	559.42	1737.07	↓**	↑**	< 0.001	< 0.001
PGA1	15548.11	34.33	107.29	↓**	↑**	< 0.001	< 0.001
Pentanoic acid	72.15	511.75	128.42	↑**	↓**	0.002	< 0.001
Pantetheine	1260.32	4753.35	1419.72	↑**	↓**	< 0.001	< 0.001
NPC	835.66	4287.74	963.52	↑**	↓**	< 0.001	< 0.001
*N*_*2*_‐hydroxyguanosine 5′‐monophosphate	0.02	3332.06	34.43	↑**	↓**	< 0.001	< 0.001
Myristoleic acid	303.87	3510.31	996.34	↑**	↓**	< 0.001	< 0.001
Methanesulfonic acid	85.51	1172.40	109.20	↑**	↓**	< 0.001	< 0.001
*L*‐gamma‐Glutamyl‐L‐alanine	1234.80	26283.55	3276.31	↑**	↓**	< 0.001	< 0.001
Indole‐3‐ethanol	574.92	73.96	154.27	↑**	↓**	< 0.001	< 0.001
Icosenoyl‐CoA	1238.75	107.58	433.85	↓**	↑**	< 0.001	< 0.001
Gln‐KPA	813.11	176.02	570.56	↓**	↑**	< 0.001	< 0.001
Fructoselysine‐6‐phosphate	0.00	579.99	18.48	↑**	↓**	< 0.001	< 0.001
Ethyl lactate	2398.83	8564.10	2894.12	↑**	↓**	< 0.001	< 0.001
Enterodiol	13725.01	152003.74	36046.94	↑**	↓**	< 0.001	< 0.001
Cis‐3‐(3‐Carboxyethenyl)‐3,5‐cyclohexadiene‐1,2‐diol	0.00	537.98	66.23	↑**	↓**	< 0.001	< 0.001
All‐Trans‐3,4‐Didehydro Retinol	46.51	266.23	67.64	↑**	↓**	< 0.001	< 0.001
9‐cis‐retinol	1997.31	8632.27	3051.87	↑**	↓**	< 0.001	< 0.001
7,8‐Dihydro‐7,8‐dihydroxykynurenate	459.52	2174.41	740.90	↑**	↓**	< 0.001	< 0.001
6,7‐Dimethyl‐8‐(1‐D‐ribityl)lumazine	302.05	1887.28	504.16	↑**	↓**	< 0.001	< 0.001
5′‐Phosphoribosylglycinamide	145.22	3971.22	173.92	↑**	↓**	< 0.001	< 0.001
4‐Hydroxycinnamic acid	149.11	563.27	161.16	↑**	↓**	< 0.001	< 0.001
4‐(Glutamylamino) butanoate	0.01	3208.81	0.27	↑**	↓**	< 0.001	< 0.001
3‐Dehydrosphinganine	10.56	457.74	95.14	↑**	↓**	0.002	< 0.001
3′,5′‐cyclic dAMP	1.18	1140.74	133.12	↑**	↓**	< 0.001	< 0.001
2‐Aminobenzoic acid	27.26	346.25	97.76	↑**	↓**	< 0.001	< 0.001
2‐Amino‐3‐carboxymuconic acid semialdehyde	8.48	220.48	71.52	↑**	↓**	< 0.001	< 0.001
(*S*)‐3‐hydroxyhexacosanoyl‐CoA	10.68	1243.33	110.80	↑**	↓**	< 0.001	< 0.001

** denote *p* < 0.01.

**FIGURE 10:**
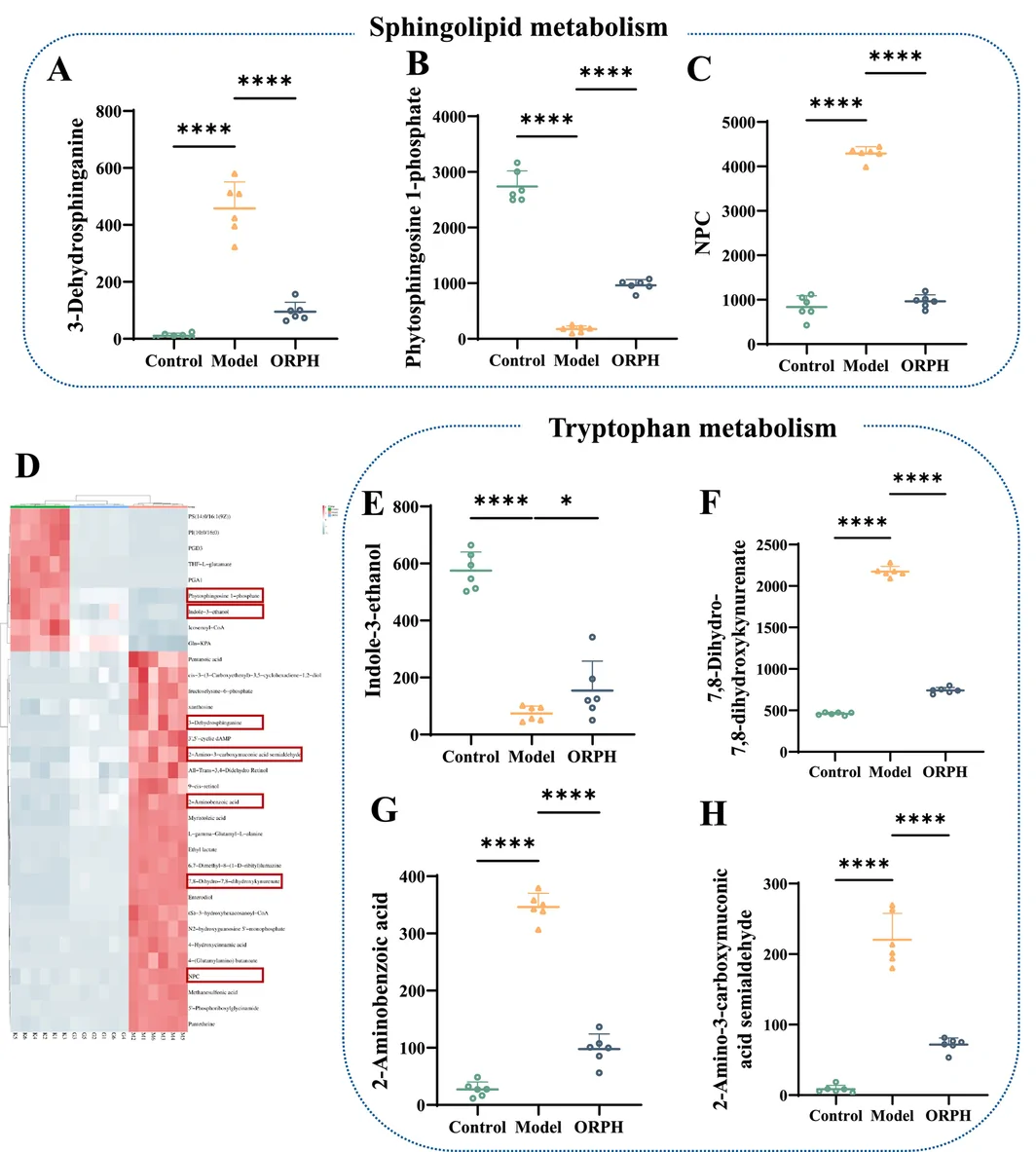
Effects of ORPH on differential expression levels of key metabolites in fecal tissue from depressed mice. (A) Differential expression of 3‐Dehydrosphinganine. (B) Differential expression of Phytosphingosine 1‐phosphate. (C) Differential expression of NPC. (D) Heatmap of key metabolites. (E) Differential expression of Indole‐3‐ethanol. (F) Differential expression of 7,8‐dihydro‐7,8‐dihydroxykynurenate. (G) Differential expression of 2‐aminobenzoic acid. (H) Differential expression of 2‐amino‐3‐carboxymuconic acid semialdehyde. *, and **** denote *p* < 0.05, and 0.0001, respectively, compared with the Model group.

### 3.8 Effects of ORPH on Mice in Pathway Blockade Verification Experiments

Previous studies suggest that ORPH may ameliorate depressive‐like phenotypes, possibly in association with gut microbiota‐dependent tryptophan and sphingolipid metabolism. Therefore, the present study performed reverse validation by establishing three intervention models: gut microbiota depletion with antibiotics (ABX), PCPA treatment to inhibit tryptophan hydroxylase (TPH) activity, and SKI‐II administration to target sphingosine kinase (SphK), to verify whether ORPH alleviates depressive symptoms through gut microbiota regulation. The detailed experimental workflow is presented in Figure 11A.

**FIGURE 11:**
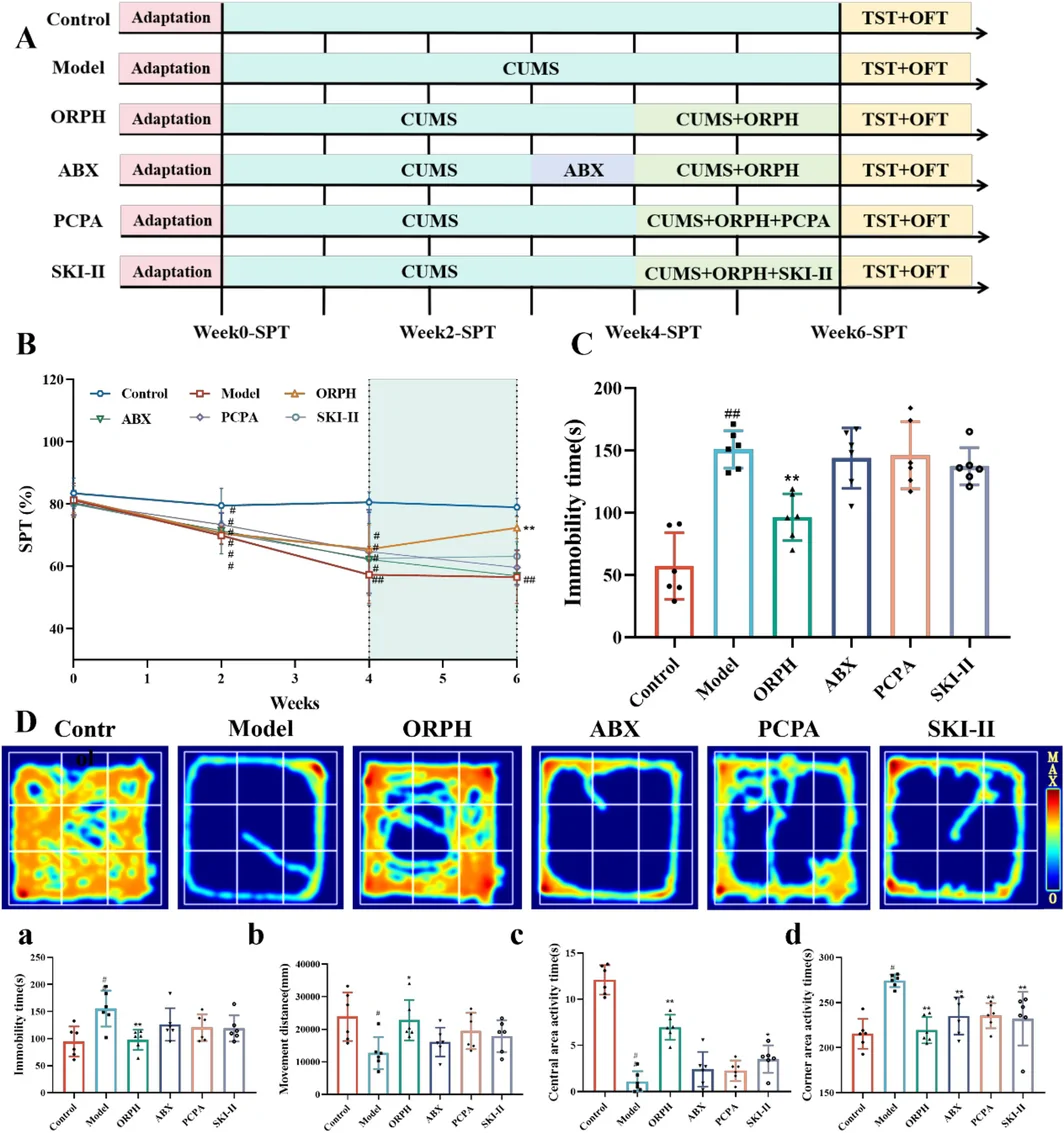
Behavioral alterations in mice after pathway blockade. (A) Schematic of experimental design. (B) Sucrose preference ratio. (C) Immobility time in the TST. (D) OFT locomotor trajectory plot: (a) immobility time, (b) movement distance, (c) central activity time, (d) corner activity time. ## denote *p* < 0.01 compared with the Control group; ** denote *p* < 0.01 compared with the Model group.

Behavioral test results (Figure 11B–D) revealed that compared with the Model group, ORPH treatment markedly elevated the sucrose preference rate, shortened immobility time in TST and OFT, increased total movement distance and central zone activity time, and decreased corner zone activity time. However, the antidepressant behavioral improvements induced by ORPH were completely abrogated in the ABX, PCPA, and SKI‐II groups relative to the standalone ORPH group.

Meanwhile, serum and brain tissue biochemical indicators of mice in each group were quantified using ELISA. ORPH significantly downregulated the levels of IL‐1β, TNF‐α, Kyn, and Cer, while upregulating cerebral Trp, 5‐HT, and S1P concentrations in CUMS‐induced depressive mice. In contrast to the ORPH group, all biochemical alterations induced by ORPH were reversed in the ABX, PCPA, and SKI‐II intervention groups. Blockade treatments with ABX, PCPA, or SKI‐II significantly abolished the ORPH‐mediated reductions in IL‐1β, TNF‐α, Kyn, and Cer, as well as the ORPH‐triggered elevations in Trp, 5‐HT, and S1P. Notably, PCPA treatment markedly increased cerebral Kyn content and reduced brain 5‐HT levels, whereas SKI‐II administration fully reversed the regulatory effects of ORPH on cerebral Cer and S1P levels. Relevant data are shown in (Figure 12A–E and Figure S4A–D).

**FIGURE 12:**
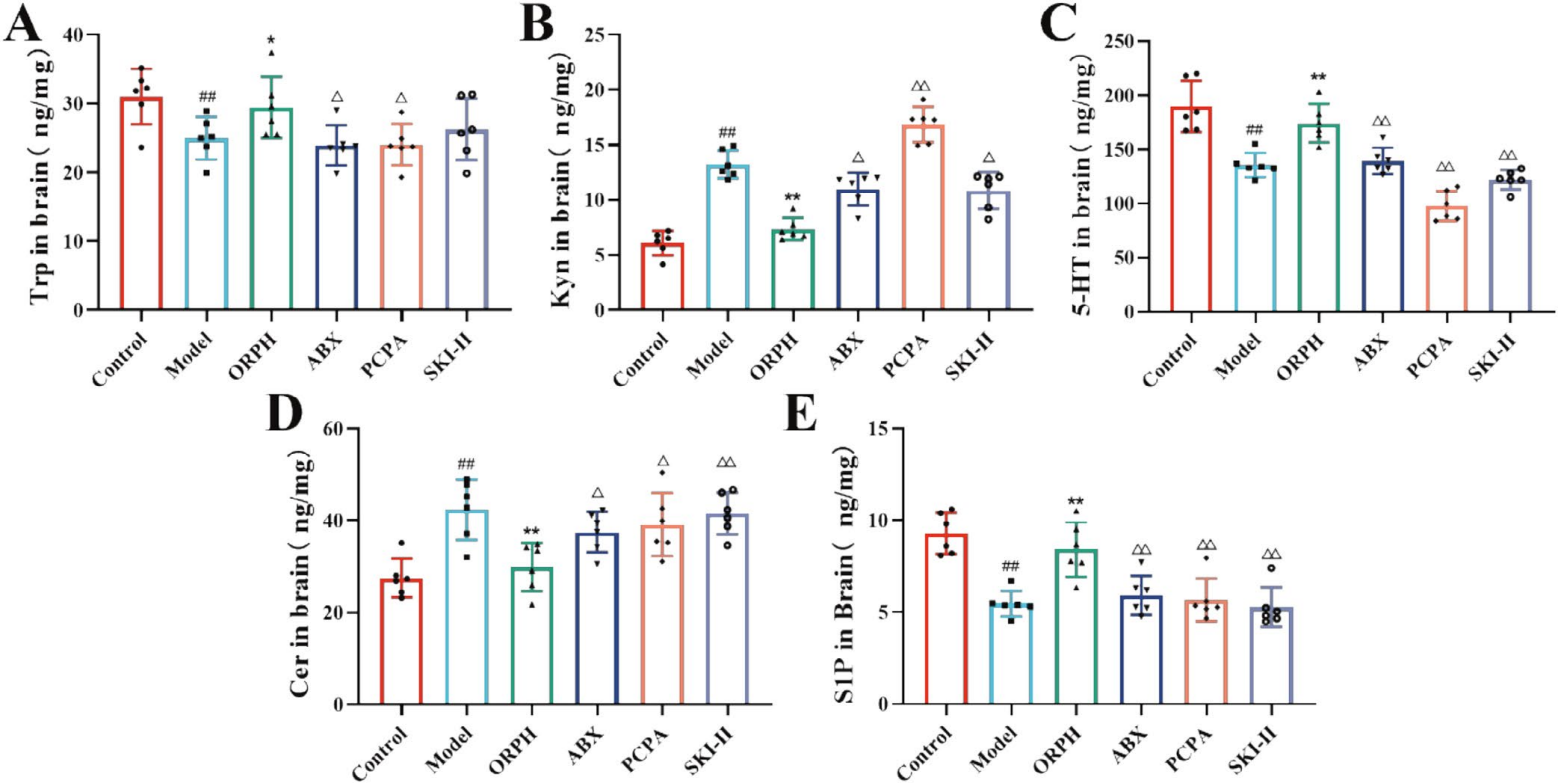
Alterations in cerebral biochemical parameters following pathway blockade. (A) Trp levels in brain tissue. (B) Kyn levels in brain tissue. (C) 5‐HT levels in brain tissue. (D) Cer levels in brain tissue. (E) S1P levels in brain tissue. ## denote *p* < 0.01 compared with the Control group, respectively; * and ** denote *p* < 0.05 and 0.01 compared with the Model group; △ and △△ denote *p* < 0.05 and 0.01 compared with the ORPH group, respectively.

## 4 Discussion

This study confirms the efficacy of ORPH in ameliorating depression. The CUMS‐induced depression model is a well‐established preclinical tool that mimics certain core symptoms of human depression, including anhedonia and behavioral despair, and is therefore frequently employed to screen potential novel therapeutics. The CUMS‐induced depressive model directly influences behavioral alterations, neuroinflammation, and neurotransmitter levels in mice. It also induces hippocampal tissue damage and neuronal apoptosis within the brain, while simultaneously causing significant alterations in gut structure and function. These changes further impact the central nervous system via the gut‐brain axis. Research indicates that CUMS‐induced depressive‐like behavior directly triggers gut microbiota dysbiosis, which in turn exacerbates neuroinflammation, establishing a bidirectional vicious cycle between depressive behavior and inflammation (Hao et al. 2024; Li et al. 2025). Our experiments demonstrate that ORPH effectively modulates gut microbiota, suppresses systemic inflammation, and alleviates depressive‐like behavior in mice. Its mechanism primarily involves downregulating pro‐inflammatory factors (IL‐6, TNF‐α, IL‐1β), regulating key metabolic pathways, and balancing gut microbial composition, thereby exerting antidepressant effects through a triple pathway.

Most current CUMS antidepressant intervention studies are based on plant extracts, with tryptophan metabolism regarded as the major downstream effector pathway (Li et al. 2026; Wang et al. 2023). In the present study, we took ORPH as the research object and integrated gut microbiome, fecal metabolome, and brain untargeted metabolome analyses, demonstrating that ORPH exerts pleiotropic regulatory effects.

Experiments demonstrate that CUMS‐induced stress markedly alters the behavior of normal mice, causing hyperactivity of the HPA axis and sustained elevation of CORT levels. This leads to disrupted stress regulation, gut microbiota dysbiosis, and excessive expression of inflammatory cytokines IL‐6, IL‐1β, and TNF‐α in serum. Concurrently, the blood–brain barrier is compromised, allowing inflammatory mediators to cross into the brain, where significant neuroinflammation is also observed in brain tissue. Following ORPH treatment, excessive expression of inflammatory factors including IL‐6, IL‐1β, and TNF‐α in serum and brain tissue was markedly suppressed. Serum ACTH and CRH levels, alongside brain CORT levels, were downregulated, while brain 5‐HT and NE levels increased. Research indicates that gut microbiota dysbiosis is a key driver of neuroinflammation, which in turn remodels the brain's metabolic environment (Wu and Zhang 2023).

We further applied non‐targeted metabolomics to identify characteristic metabolic pathway abnormalities associated with depression, particularly disturbances in sphingolipid and tryptophan metabolism. These metabolic imbalances are directly linked to the onset and progression of depression. Metabolomics technology offers a unique molecular perspective for deciphering the pathological mechanisms of depression. By systematically detecting dynamic changes in endogenous metabolites, it reveals the interplay between inflammation and metabolic disorders (Zarei et al. 2025). Our pathway enrichment analysis of differentially expressed metabolites in brain tissue identified sphingolipid metabolism as a key pathway. Inflammation triggers sphingolipid metabolic disruption in brain tissue; elevated TNF‐α levels in depressed mice activate neutral sphingolipidase, leading to IL‐1β release and exacerbating neuronal damage (Al‐Rashed et al. 2020). Our findings demonstrate that GM3 (d18:1/9Z‐18:1), Galabiosylceramide (d18:1/9Z‐18:1), and *N*, *N*‐Dimethylsphingosine were significantly elevated. The abnormal accumulation of these metabolites is closely associated with neuroinflammation and apoptosis (Hülsmeier 2025). Following ORPH treatment, the levels of these disrupted metabolites were significantly reversed and normalized. We hypothesize that ORPH's therapeutic effect may regulate the sphingolipid metabolic pathway by directly inhibiting inflammatory factors and restoring gut‐brain axis function.

The gut microbiota and its metabolites, as core elements regulating the gut‐brain axis, influence brain function and nervous system modulation through multiple mechanisms. Furthermore, gut microbes not only directly affect the synthesis and metabolism of key neurotransmitters such as 5‐HT and NE, but also play a significant role in regulating neuroinflammation (Bhatia et al. 2023; Strandwitz 2018). Recent studies have revealed the gut‐brain axis's pivotal role in depression treatment. Clinical evidence indicates that modulating gut microbiota composition through probiotic interventions or fecal microbiota transplantation significantly alleviates depressive symptoms (Liu, Wang, et al. 2022; Zhou et al. 2023). This study integrates 16S rRNA sequencing with fecal metabolomics to elucidate the mechanism by which ORPH alleviates depression through regulating gut dysbiosis. Results indicate that differentially abundant metabolites in fecal tissue predominantly enrich three pathways: Purine metabolism, Sphingolipid metabolism, and Tryptophan metabolism. Evidence indicates that altered microbial composition may lead to excessive activation of pro‐inflammatory factors (such as IL‐6 and TNF‐α), directly modifying the intestinal sphingolipid metabolic environment (Norris and Blesso 2017). Concurrently, gut microbiota, as participants in tryptophan metabolism, influence this pathway through multiple mechanisms (Hou et al. 2023; Liu, Nieuwdorp, et al. 2022). Notably, abnormal accumulation of sphingolipid metabolites can activate microglia, prompting the release of inflammatory cytokines that feedback‐regulate metabolic states, thereby establishing a vicious ‘inflammation‐metabolism’ cycle (Blank et al. 2022; Lu et al. 2021). This study observed significantly elevated levels of 7,8‐Dihydro‐7,8‐dihydroxykynurenate, 2‐Aminobenzoic acid, and 2‐Amino‐3‐carboxymuconic acid semialdehyde, while Indole‐3‐ethanol levels were markedly reduced. This may correlate with the substantial increase in pro‐inflammatory factors and the suppression of gut indole‐producing bacterial abundance.

At present, research has confirmed that the microbial composition within individuals with depression exhibits significant alterations compared to healthy populations, suggesting that modulating the gut microbiota may prove beneficial for depression treatment (Liu, Wang, et al. 2022). At the gut microbiota level, ORPH specifically enhances species richness rather than evenness, indicating its selective regulatory effect on microbial niches; concurrently, β‐diversity analysis demonstrates that ORPH significantly remodels CUMS‐induced disruptions in microbial community structure. Research indicates that *Bacillota* and *Bacteroidot* are the phyla most profoundly affected by depression (Zheng et al. 2020). Our findings demonstrate reduced *Bacteroidot* and markedly increased *Bacillota* in depressed mice. Following ORPH intervention, the abundance of both phyla markedly reversed, shifting the *Bacillota/Bacteroidota* ratio toward that of control mice and supporting modulation of the gut microbiota. In the Model group, we observed increases in the relative abundance of *Enterococcus* and *Desulfovibrionaceae*. The former exacerbates inflammation‐induced intestinal barrier damage (Chen et al. 2021), while the latter generates neurotoxic hydrogen sulfide through sulfate reduction (Murros 2022), collectively promoting neuroinflammation. ORPH intervention reshaped the gut microbiota structure in depressed mice, establishing an ecosystem dominated by *Lactobacillus*. We observed that ORPH attenuated central nervous system inflammation. Previous studies propose that such alterations in the gut microbial ecosystem may alleviate central nervous system inflammation via vagal transmission and the circulatory system (Li, Peng, et al. 2023; Wang et al. 2024). Concurrently, *Lactobacillus*, as an indole‐producing bacterium, promotes the conversion of tryptophan to 5‐HT and inhibits the kynurenine pathway, earning it the designation of a “psychoactive probiotic” (Haq et al. 2021). Of particular note, residual *Desulfovibrio* may indicate partial resistance to intervention within this genus, yet its abundance remained lower than in the Model group. Specifically, ORPH reduced the abundance of taxa reported to harbor pro‐inflammatory potential (e.g., *Clostridium*, *Enterococcus*) (Li, Cheng, et al. 2024; Liu et al. 2024), which may contribute to lowered systemic inflammation, alleviated tryptophan metabolic disorders, and increasd 5‐HT synthesis in the brain. Meanwhile, ORPH increased the abundance of anti‐inflammatory microbes (e.g., *Bacteroidetes*), elevating anti‐inflammatory factor levels, while reducing NE release in brain tissue, thereby aiding mood recovery in depressed mice. The enrichment of *Akkermansia* and *Bifidobacterium* play a pivotal role (Li, Wei, et al. 2024). *Akkermansia* enhances intestinal barrier function by thickening the mucus layer (Pan et al. 2025), thereby reducing systemic inflammatory responses. Its metabolite propionate is hypothesized to cross the blood–brain barrier, inhibiting microglial activation (downregulating IL‐6 and TNF‐α expression) and alleviating neuroinflammation (Wang et al. 2021). As previously hypothesized, *Bifidobacterium* can competitively consume tryptophan substrates via its unique tryptophan metabolic pathway, increasing peripheral 5‐HT reserves and competitively inhibiting the IDO pathway. This reduces the production of neurotoxic kynurenine metabolites, achieving a ‘directed diversion’ of tryptophan (Li, Wang, et al. 2023).

These findings support an association between specific microbiota and depressive phenotypes. ORPH treatment was accompanied by a shift in gut microbiota composition, characterized by lower relative abundance of *Enterococcus* and *Desulfovibrionaceae* and higher relative abundance of *Lactobacillus*. These microbial alterations, alongside the diversion of tryptophan metabolism toward the 5‐HT synthesis pathway, the restoration of sphingolipid metabolic homeostasis, and the alleviation of neuroinflammation, collectively constitute the multi‐targeted action mechanism of ORPH. To explore whether the antidepressant effect of ORPH is dependent on the regulation of gut microbiota, tryptophan metabolism, and sphingolipid metabolism, we depleted the gut microbiota using ABX, inhibited TPH activity with PCPA, and inhibited SphK using SKI‐II. All three interventions reversed the therapeutic effect of ORPH on depression. ELISA analysis of key metabolites revealed that ABX, PCPA, and SKI‐II also reversed the ORPH‐induced regulation of Trp, Kyn, 5‐HT, Cer, and S1P levels. Notably, PCPA significantly increased Kyn levels and decreased 5‐HT levels in brain tissue, which is consistent with the classic mechanism of tryptophan metabolism in which 5‐HT synthesis is blocked and the metabolic flux is redirected toward the kynurenine pathway. This finding further suggests that ORPH may exert its antidepressant effect by modulating the tryptophan metabolic pathway. Meanwhile, SKI‐II reversed the regulatory effect of ORPH on Cer and S1P levels in brain tissue, further indicating that ORPH alleviates depressive‐like behaviors in CUMS mice through the regulation of the sphingolipid metabolic pathway. This study not only provides novel experimental evidence for un708derstanding the gut‐brain axis mechanisms in depression but also lays a theoretical foundation for developing microbiome‐regulated antidepressant strategies.

However, only male mice were used to establish the CUMS model in this study, and the effects of ORPH in female animals remain to be systematically verified. Meanwhile, although the ABX depletion and pathway‐blockade experiments imply an association between ORPH and the gut microbial ecosystem, fecal microbiota transplantation (FMT) assays were not performed, and thus a direct causal link between ORPH and gut microbiota cannot be established. In addition, after conversion based on body surface area, the ORPH dose of 0.2–0.8 g/kg in mice approximates 21.9–87.6 mg/kg in humans, equivalent to 1.31–5.26 g/day for a 60 kg adult. This dosage range is comparable to commonly used doses of dietary protein hydrolysates and peptide supplements, supporting the feasibility of oral administration. Nevertheless, issues regarding ORPH purity, batch standardization, and production cost need to be addressed prior to human studies.

## 5 Conclusions

In summary, ORPH ameliorated CUMS‐induced depressive‐like behaviors in mice, attenuated serum and cerebral pro‐inflammatory cytokines, normalized HPA‐axis hormones, restored cerebral 5‐HT and NE levels, and alleviated hippocampal neuronal injury and apoptosis. These improvements were accompanied by altered gut microbiota composition, fecal metabolites, and brain metabolites, with enrichment of pathways related to tryptophan and sphingolipid metabolism. In the pathway‐blockade experiment, the effects of ORPH were attenuated by ABX, PCPA, and SKI‐II. This discovery not only provides a robust theoretical basis for ORPH's efficacy in ameliorating depression‐associated depressive‐like behaviors but also underscores the pivotal role of the gut microbiota in depression intervention therapies.

## Author Contributions

**Mengmeng Liu:** methodology, visualization, writing – original draft. **Jingyi Niu:** methodology. **Yukang Miao:** methodology. **Wentao Guo:** validation, methodology. **Xiaowei Huang:** supervision. **Hongyan Chen:** methodology. **Zhe Lin:** supervision, conceptualization. **Guangfu Lv:** conceptualization, writing – review and editing.

## Funding

This research was funded by Jilin Provincial Scientific and Technological Development Program, grant number YDZJ202501ZYTS770.

## Ethics Statement

The animal study protocol was approved by the Ethics Committee of Changchun University of Chinese Medicine.

## Conflicts of Interest

The authors declare no conflicts of interest.

## Supporting information


**Figure S1:** Escape latency during the training phase (days 3–5) of the MWM.
**Figure S2:** Permutation test of the OPLS‑DA model. (A) Permutation test of Control versus Model group. (B) Permutation test of Model versus ORPH groups.
**Figure S3:** Correlation analysis. The color scale represents *p* values, with darker color indicating smaller *p* values.
**Figure S4:** Alterations in serum and cerebral biochemical parameters following pathway blockade. (A) Serum TNF‐α levels. (B) Serum IL‐1β levels. (C) TNF‐α levels in brain tissue. (D) IL‐1β levels in brain tissue. ## denote *p* < 0.01 compared with the Control group, respectively; ** denote *p* < 0.01 compared with the Model group; △△ denote *p* < 0.01 compared with the ORPH group, respectively.
**Figure S5:** Behavioral alterations in mice after pathway blockade. (A) Schematic of experimental design. (B) Sucrose preference ratio. (C) Immobility time in the TST. (D) OFT locomotor trajectory plot: (a) immobility time, (b) movement distance, (c) central activity time, (d) corner activity time. # and ## denote *p* < 0.05 and 0.01 compared with the Control group, respectively; * and ** denote *p* < 0.05 and 0.01 compared with the Model group, respectively.
**Table S1:** Analytical performance parameters of commercial ELISA kits.
**Table S2:** Results of fixed‐effect tests in the linear mixed‐effects model.
**Table S3:** Peptide information identified by PEAKS Studio (Top 10 scored peptides).

## Data Availability

The data that support the findings of this study are available on request from the corresponding author.
