# Supplementary material for: Oviductus Ranae Protein Hydrolysate (ORPH) Ameliorates CUMS‐Induced Depressive‐Like Behaviors in Mice via Modulation of Gut Microbiota and Metabolic Signatures: An Integrated Multi‐Omics Investigation
Source: Food Sci Nutr. 2026 Nov 13;14(11):e72572. doi: 10.1002/fsn3.72572 (PMC13768601; doi:10.1002/fsn3.72572)
Supplement: Supplementary file 1 [file FSN3-14-e72572-s001.docx]

**Supplementary material**

***Oviductus Ranae* protein hydrolysate (ORPH) ameliorates CUMS-induced depressive-like behaviors in mice via modulation of gut microbiota and metabolic signatures: an integrated multi-omics investigation**

Mengmeng Liu,^a^ Jingyi Niu,^a^ Yukang Miao,^a^ Wentao Guo,^a^ Xiaowei Huang,^a^ Hongyan Chen,^b^ Zhe Lin ^a*^ and Guangfu Lv ^a*^

a Changchun University of Chinese Medicine, Changchun, China

b The Affiliated Hospital to Changchun University of Chinese Medicine, Changchun, China

* Correspondence: LV: [lvgf@ccucm.edu.cn](mailto:lvgf@ccucm.edu.cn) LIN: [linzhe@ccucm.edu.cn](mailto:linzhe@ccucm.edu.cn)

* Corresponding author:

Zhe Lin, Changchun University of Chinese Medicine, Changchun 130117, China; e-mail: [linzhe@ccucm.edu.cn](mailto:linzhe@ccucm.edu.cn)

Guangfu Lv, Changchun University of Chinese Medicine, Changchun 130117, China; e-mail: [lvgf@ccucm.edu.cn](mailto:lvgf@ccucm.edu.cn)

**The specific methods of Nano-LC-MS/MS**

Non-targeted peptidomic analysis was performed using nano-LC-MS equipped with an electrospray ionisation (ESI) source. Liquid chromatography separation was conducted on a nano-flow chromatographic system consisting of a pre-column (150 μm × 50 mm, Reprosil-Pur 120 C18-AQ, 3 μm) and an analytical column (150 μm × 170 mm, Reprosil-Pur 120 C18-AQ, 1.9 μm).

Specific chromatographic parameters: Mobile phase A was 0.1% formic acid aqueous solution, mobile phase B was 80% acetonitrile containing 0.1% formic acid, gradient elution programme (0 min, 4% B; 2 min, 8% B; 45 min, 28% B; 55 min, 40% B; 56-66 min, 95% B), flow rate 600 nL·min^-1^, total analytical runtime 66 min.

Mass spectrometry parameters were set as follows: full-scan MS1 resolution of 70,000 with an AGC target of 3×10^6^ and maximum injection time of 100 ms across the m/z range of 100-1500; data-dependent MS2 fragmentation was operated at a resolution of 17,500, an AGC target of 1×10^5^ and maximum injection time of 50 ms, with top 20 precursor ions selected for fragmentation at a normalized collision energy of 28. Raw datasets were searched against uniprotkb reference database with specified fixed/variable modifications and mass tolerance thresholds.

Raw mass spectrometry data were searched using PEAKS software with a false discovery rate (FDR) of 1% for both peptide and protein identification. The database search parameters were set as follows: carbamidomethylation of cysteine (C) was defined as a fixed modification, while oxidation of methionine (M) and N-terminal acetylation of peptides were assigned as variable modifications. Non-specific cleavage was selected for enzyme digestion. The mass tolerance was set to 20 ppm for precursor ions (MS1) and 0.02 Da for fragment ions (MS2). All spectra were searched against the UniProt database (uniprotkb_taxonomy_id_8399_2025_02_07.fasta). The software output yielded PSM-10lgP, a significance score for peptides identified via database searching or de novo sequencing, which was presented as “Score” in Table S3.

Animal experimental design

After acclimatization, mice were stratified by body weight and then randomly assigned to experimental groups (n = 10 or 6 per group). Random sequences were generated using the Excel RAND function. The CUMS model was established according to the experimental design, and the corresponding drugs (e.g., fluoxetine and ORPH) were administered by gavage. Behavioral assessments were performed by investigators blinded to group allocation. Behavioral tests were conducted in the order of OFT, MWM, TST, and FST, with a 48-h interval between tests. H&E and TUNEL staining results were evaluated by an investigator blinded to the experimental procedures and group allocation. The MWM training lasted for 5 days in total. The first 2 days served as the adaptation phase, in which mice were trained in clear water, and data collected during this period were not subjected to statistical analysis. Formal training was performed on days 3-5 with opaque water. Each mouse received training trials across four quadrants, and escape latency was recorded. The corresponding results are shown in Figure S1.

Characteristics of Elisa Kits

The analytical performance of each commercial ELISA kit from Jiangsu Enzyme Immunoassay is documented in the product instructions, with detailed parameters shown in Table S1.

Quality Control and Data Processing for Untargeted Metabolomics

Hippocampal and fecal samples from six randomly selected mice per group were collected for metabolomic profiling. To ensure data reliability, pooled quality control (QC) samples were prepared by mixing equal volumes of all samples and injected every three to four samples throughout the analytical run. The QC samples clustered tightly in the PCA score plot, with pairwise correlation coefficients > 0.8. Given the untargeted metabolomics approach, the proportion of peaks with a relative standard deviation (RSD) ≤ 30% among the total identified peaks (RSD_Ratio) was employed as a dataset-level metric to evaluate analytical repeatability. The RSD_Ratio of the QC samples was ≥ 0.6, indicating high repeatability and instrument stability. 2-Chloro-L-phenylalanine (20 mg/L; Aladdin, Shanghai, China) was added as an internal standard. Metabolite peak intensities were normalized using Progenesis QI software. Missing values were imputed using one-tenth of the minimum positive value. All samples were analyzed within a single batch. Inspection of the QC sample PCA score plots revealed no systematic drift associated with injection order; thus, no additional batch effect correction was required.

**Supplementary Pathway Blocking Validation Assays**

54 SPF male ICR mice, weighing 18-22 g, were purchased from Liaoning Changsheng Biotechnology Co., Ltd. (Jilin, China). The feeding environment was the same as previously described. After one week of acclimatization, the mice were randomly divided into Control, CUMS, CUMS+ORPH, CUMS+ABX, CUMS+PCPA, CUMS+SKⅠ-Ⅱ, Control+ABX, Control+PCPA, and Control+SKⅠ-Ⅱ groups, with 6 mice in each group. Except for the Control, Control+ABX, Control+PCPA, and Control+SKⅠ-Ⅱ groups, a depression mouse model was established using CUMS. All stress stimuli started from the first week, with no two consecutive stimuli being the same, and the stimulation lasted for 28 days. In the CUMS+ABX and Control+ABX groups, a broad-spectrum antibiotic mixture was added to the drinking water from day 14 to day 28 to deplete the gut microbiota. Drug administration began on day 14. Mice in the CUMS+ORPH group were administered ORPH at a dose of 0.8 g/kg via oral gavage, while those in all other groups received an equal volume of normal saline through the same route. The CUMS+PCPA and Control+PCPA groups received an intraperitoneal injection of 250 mg/kg PCPA, and the CUMS+SKⅠ-Ⅱ and Control+SKⅠ-Ⅱ groups received an intraperitoneal injection of 50 mg/kg SKⅠ-Ⅱ. The detailed procedure is shown in Figure S5A. The SPT was conducted every two weeks. After the 4th week, behavioral experiments were performed. Following the behavioral tests, the mice were euthanized under anesthesia.

We established a CUMS-induced depression mouse model and subsequently administered ABX (for gut microbiota depletion), intraperitoneal PCPA (to inhibit TPH activity), and intraperitoneal SKI-II (to inhibit SphK activity) to investigate whether these interventions exacerbate depressive-like behaviors. The same interventions were applied to non-stressed control mice to determine whether they could directly induce depressive-like behaviors. Behavioral tests (SPT, TST, and OFT) revealed that treatment with ABX, PCPA, or SKI-II alone did not significantly alter depressive-like behaviors in either CUMS or control mice. The corresponding results are presented in Figure S5A-D.

**Figure S1.** Escape latency during the training phase (days 3-5) of the MWM.

**Figure S2.** Permutation test of the OPLS‑DA model. (**A**) Permutation test of Control versus Model group. (**B**) Permutation test of Model versus ORPH groups.

**Figure S3.** Correlation analysis. The color scale represents *P* values, with darker color indicating smaller *P* values.

**Figure S4.** Alterations in serum and cerebral biochemical parameters following pathway blockade. (**A**) Serum TNF-α levels. (**B**) Serum IL-1β levels. (**C**) TNF-α levels in brain tissue. (**D**) IL-1β levels in brain tissue. ## denote *P* < 0.01 compared with the Control group, respectively; ** denote *P* < 0.01 compared with the Model group; △△ denote *P* < 0.01 compared with the ORPH group, respectively.

**Figure S5.** Behavioral alterations in mice after pathway blockade. (**A**) Schematic of experimental design. (**B**) Sucrose preference ratio. (**C**) Immobility time in the TST. (**D**) OFT locomotor trajectory plot: **a**. immobility time, **b**. movement distance, **c**. central activity time, **d**. corner activity time. # and ## denote *P* < 0.05 and 0.01 compared with the Control group, respectively; * and ** denote *P* < 0.05 and 0.01 compared with the Model group, respectively.

**Table S1: Analytical performance parameters of commercial ELISA kits**

|  | Assay Type | Standard Curve R | Detection Range | Sensitivity (LOD) | Intra/Inter CV |
| --- | --- | --- | --- | --- | --- |
| S1P | Competitive | ≥0.9900 | 12.5-200 ng/mL | <1 ng/mL | <15% |
| Kyn |  |  | 125-2000 pmol/mL | <10 pmol/mL |  |
| Trp |  |  | 10-160 ng/mL | <1 ng/mL |  |
| Cer |  |  | 50-800 pg/mL | <1 pg/mL |  |
| 5-HT | Sandwich | ≥0.95 | 8-240 pg/mL | ~8 pg/mL | <10%/<12% |
| IL-1β |  |  | 2.5-80 ng/L | ~2.5 ng/L |  |
| IL-6 |  |  | 3-120 pg/mL | ~3 pg/mL |  |
| CRH |  |  | 40-1200 ng/L | ~40 ng/L |  |
| ACTH |  |  | 3-120 ng/L | ~3 ng/L |  |
| CORT |  |  | 2-80 μg/L | ~2 μg/L |  |
| NE |  |  | 3-120 pg/mL | ~3 pg/mL |  |
| TNF-α |  |  | 25-800 ng/L | ~25 ng/L |  |

**Table S2: Results of fixed-effect tests in the linear mixed-effects model.**

|  | Body weight | | SPT1 | | SPT2 | | SPT3 | |
| --- | --- | --- | --- | --- | --- | --- | --- | --- |
|  | F (df_n_, df*_d_*) | P-value | F (df_n_, df*_d_*) | P-value | F (df_n_, df*_d_*) | P-value | F (df_n_, df*_d_*) | P-value |
| week | F(6, 378)=16.28 | <0.0001 | F(6, 63)=12.55 | <0.0001 | F(3, 120)=39.48 | <0.0001 | F(2, 15)=54.85 | <0.0001 |
| Group | F(4503, 340.4)=10.53 | <0.0001 | F(4594, 289.4)=60.05 | <0.0001 | F(2579, 61.89)=11.99 | <0.0001 | F(4.842, 72.63)=16.70 | <0.0001 |
| week×Group | F(30, 378)=2.02 | 0.0017 | F(30, 315)=10.62 | <0.0001 | F(15, 120)=1.986 | 0.0207 | F(16, 120)=6.96 | <0.0001 |

Df_n_, numerator degrees of freedom; df*_d_*, denominator degrees of freedom.

**Table S3: Peptide information identified by PEAKS Studio (Top 10 scored peptides)**

| **Protein Accession** | **Peptide** | **Score** | **Length** | **ppm** | **m/z** | **z** | **RT** | **Area** |
| --- | --- | --- | --- | --- | --- | --- | --- | --- |
| M9NWM2\|M9NWM2_9NEOB:M9NWY2\|M9NWY2_RANBO:A0A8K0Y7I3\|A0A8K0Y7I3_9NEOB | GQKDSYVGDEAQSKRGIL | 59.79 | 18.0 | 1.4 | 651.00171 | 3.0 | 24.6785 | 9.29E5 |
| M9NWM2\|M9NWM2_9NEOB:M9NWY2\|M9NWY2_RANBO:A0A8K0Y7I3\|A0A8K0Y7I3_9NEOB | GQKDSYVGDEAQSKRGILT | 57.53 | 19.0 | 2.6 | 513.76569 | 4.0 | 23.5141 | 6.31E5 |
| M9NWM2\|M9NWM2_9NEOB:M9NWY2\|M9NWY2_RANBO:A0A8K0Y7I3\|A0A8K0Y7I3_9NEOB | GQKDSYVGDEAQSKR | 56.56 | 15.0 | 1.5 | 556.6051 | 3.0 | 10.509 | 2.27E4 |
| A0A8K0Y7I3\|A0A8K0Y7I3_9NEOB | GFAGDDAPRAVFPS | 55.45 | 14.0 | 2.2 | 703.84009 | 2.0 | 41.6158 | 9.87E5 |
| P13105\|TPM1_RANTE | EAKHIAEEADRKYEEVARKL | 55.23 | 20.0 | 3.0 | 477.85767 | 5.0 | 30.4316 | 5.92E5 |
| A0A0U1T4U8\|A0A0U1T4U8_RANTE | KGEHSIIGRTA | 53.59 | 11.0 | 1.8 | 584.82629 | 2.0 | 11.9128 | 6.52E5 |
| P13105\|TPM1_RANTE | M(+42.01)DAIKKKMQML | 48.57 | 11.0 | 3.6 | 689.8689 | 2.0 | 49.7011 | 2.26E6 |
| A0A8K0Y7I3\|A0A8K0Y7I3_9NEOB | GFAGDDAPRAVFPSIVGRP | 47.49 | 19.0 | 3.2 | 643.67279 | 3.0 | 49.0284 | 6.06E5 |
| F5BBG7\|F5BBG7_RANLU | TDGKHGFHIH | 46.9 | 10.0 | 1.8 | 574.78442 | 2.0 | 10.6068 | 2.28E6 |
| A0A8K0Y7I3\|A0A8K0Y7I3_9NEOB | GFAGDDAPRAVFPSIVGRPRHQGVMVGM | 44.2 | 28.0 | 1.7 | 731.87592 | 4.0 | 46.7374 | 2E6 |
